# Multi‐Omics Reveal the Dysregulated Gut‐Joint Axis in Knee Synovitis: Data from Two Osteoarthritis Studies in China

**DOI:** 10.1002/advs.202512020

**Published:** 2025-12-07

**Authors:** Xiaoshuai Wang, Yi Liu, Ziying Sun, Jia Li, Zhihao Lu, Jiayuan Huang, Shixian Hu, Peihua Cao, Xiaolong Cao, Shangru Li, Jianzhao Ruan, Jinhui Liu, Jingyu Xie, Haitao Sun, Tianyu Chen, Shengfa Li, Zhaohua Zhu, Zhibo Wen, Rocky S. Tuan, David John Hunter, Zhong Alan Li, Dongquan Shi, Changhai Ding

**Affiliations:** ^1^ Clinical Research Centre Zhujiang Hospital, Southern Medical University Guangzhou Guangdong 510280 China; ^2^ Department of Biomedical Engineering The Chinese University of Hong Kong Hong Kong 999077 China; ^3^ Department of Radiation Oncology Sun Yat‐Sen University Cancer Center State Key Laboratory of Oncology in South China Collaborative Innovation Center for Cancer Medicine Guangdong Key Laboratory of Nasopharyngeal Carcinoma Diagnosis and Therapy 651 Dongfeng Road East Guangzhou Guangdong 510060 China; ^4^ School of Medicine Shenzhen Campus of Sun Yat‐Sen University Shenzhen Guangdong 518000 China; ^5^ Department of Orthopedics Nanjing Jinling Hospital, Affiliated Hospital of Medical School Nanjing University Nanjing Jiangsu 210000 China; ^6^ Division of Orthopaedic Surgery Department of Orthopaedics Nanfang Hospital Southern Medical University Guangzhou Guangdong 510000 China; ^7^ Laboratory for Bone and Joint Disease, Division of Sports Medicine and Adult Reconstructive Surgery Department of Orthopedic Surgery Nanjing Drum Tower Hospital, Affiliated Hospital of Medical School, State Key Laboratory of Pharmaceutical Biotechnology Nanjing University 321 Zhongshan Road Nanjing Jiangsu 210008 China; ^8^ Shenzhen Key Laboratory of Systems Medicine for Inflammatory Diseases Shenzhen Campus of Sun Yat‐Sen University Shenzhen Guangdong 518000 China; ^9^ Department of Gastroenterology The First Affiliated Hospital Sun Yat‐Sen University Guangzhou Guangdong 510000 China; ^10^ Institute of Precision Medicine The First Affiliated Hospital Sun Yat‐Sen University Guangzhou Guangdong 510000 China; ^11^ Department of Anesthesiology Zhujiang Hospital Southern Medical University Guangzhou Guangdong 510280 China; ^12^ Department of Oncology The First Affiliated Hospital Sun Yat‐Sen University Guangzhou Guangdong 510000 China; ^13^ Department of Population & Public Health Sciences Keck School of Medicine University of Southern California Los Angeles CA 90089 USA; ^14^ Clinical Biobank Centre Microbiome Medicine Center Department of Laboratory Medicine Zhujiang Hospital Southern Medical University Guangzhou Guangdong 510280 China; ^15^ Neurosurgery Center Department of Cerebrovascular Surgery Engineering Technology Research Center of Education Ministry of China on Diagnosis and Treatment of Cerebrovascular Disease The National Key Clinical Specialty Guangdong Provincial Key Laboratory on Brain Function Repair and Regeneration The Neurosurgery Institute of Guangdong Province Zhujiang Hospital Southern Medical University Guangzhou Guangdong 510280 China; ^16^ Department of Orthopedics The Third Affiliated Hospital of Southern Medical University Guangzhou 510000 China; ^17^ Department of Orthopaedics The Third People's Hospital of Chengdu, Affiliated Hospital of Southwest Jiaotong University The Second Affiliated Chengdu Hospital of Chongqing Medical University Chengdu Sichuan 610000 China; ^18^ Department of Radiology Zhujiang Hospital Southern Medical University Guangzhou Guangdong 510280 China; ^19^ Department of Rheumatology Royal North Shore Hospital and Sydney Musculoskeletal Health Kolling Institute University of Sydney Sydney New South Wales 2006 Australia; ^20^ State Key Laboratory of Digestive Disease The Chinese University of Hong Kong NT Hong Kong China; ^21^ Menzies Institute for Medical Research University of Tasmania Hobart Tasmania 7005 Australia; ^22^ Clinical Research Centre Beijing Tsinghua Changgung Hospital Tsinghua Medicine Tsinghua University Beijing 102218 China

**Keywords:** knee synovitis, gut‐joint axis, multi‐omics, TWEAK, magnetic resonance imaging

## Abstract

Gut microbiota dysbiosis and associated host immuno‐metabolic disorders may play a role in knee synovitis. Herein, integrated multi‐omics analyses of stool and blood samples from subjects from Pearl River Osteoarthritis Cohort (PROC, N = 207) are conducted to explore the potential gut‐joint axis. Specifically, gut metagenomics, serum metabolomics and plasma proteomics are carried out. Knee synovitis is identified by magnetic resonance imaging. A total of 87 synovitis cases are identified in PROC, which are characterized by increased *Firmicutes*/*Bacteroidetes* (F/B) ratio. Alterations in microbial functions of both leucine and geraniol degradation are closely associated with increased serum 3‐hydroxyisovaleric acid and decreased geranic acid. These perturbations are significantly correlated with F/B ratio and down‐regulated plasma TWEAK. Building upon these, the potential synovial targets are explored using a synovial single‐cell dataset and the Nanjing Osteoarthritis Cohort (NOC, N = 22). Synovial fluid proteomics, histological analysis, and in vitro experiments with human fibroblast‐like synoviocytes (FLS) are conducted for NOC subjects with different synovitis grades. An upregulated TWEAK receptor is found in higher grade of synovitis. In vitro, higher TWEAK induced down‐regulated TWEAK receptor in FLS. The study for the first time revealed the gut‐joint axis in knee synovitis, providing new insight into potential targets for synovitis treatment.

## Graphical abstract

The “gut‐joint axis” in knee synovitis is uncovered. Integrated multi‐omics studies are conducted in two independent osteoarthritis cohorts. Synovitis is characterized an increased F/B ratio, as well as alterations of 3‐HIA, geranic acid, and TWEAK. Upregulated TWEAK receptor is found in high‐grade synovitis, and inversely correlated with lower TWEAK. This study provide new insight into the “gut‐joint axis” and reveal potential therapeutic targets.

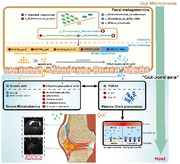

## 1 Introduction

Knee osteoarthritis (KOA) is widely accepted as a kind of low‐grade and aseptic inflammatory disorder,^[^ ^1^ ^]^ and the inflammatory phenotype is denoted as one of the major subtypes.^[^ ^2^, ^3^, ^4^, ^5^ ^]^ Moreover, growing evidence suggests that knee synovitis plays an etiopathogenic and modifiable role in KOA,^[^ ^6^, ^7^ ^]^ but the pathogenesis of synovitis is still largely unclear.

In recent years, the gut microbiota (GMB) was reported to contribute to OA,^[^ ^8^, ^9^ ^]^ since dysregulated GMB is responsible for producing metabolites that can interact with the host's innate immune system to induce further systemic inflammation.^[^ ^10^ ^]^ For instance, the altered bile acid in the host was reported as the mediator between GMB and hand synovitis,^[^ ^11^ ^]^ while dysbiotic GMB could induce lipopolysaccharides (LPS), subsequent systemic inflammation and degradation of the cartilage matrix in the host.^[^ ^12^ ^]^ Accordingly, the “gut‐joint axis” was introduced.^[^ ^13^ ^]^ However, only bacterial RNA‐sequencing (mostly 16s ribosomal RNA amplicon sequencing) or limited detection of metabolites were involved in recent studies, and the proinflammatory molecules, as well as their links to dysbiosis of GMB and metabolic disorders, remain largely unknown in knee synovitis.

It is crucial to identify patients with the inflammatory phenotype of KOA before recruiting them to the trials for drugs with anti‐inflammatory and disease‐modifying effects.^[^ ^2^, ^14^ ^]^ Of note, knee synovitis manifests as Hoffa's synovitis (i.e., inflammation in the infrapatellar fat pad), effusion synovitis, or both,^[^ ^14^, ^15^ ^]^ Nowadays, it has been proposed to take the synovium (source of effusion) and fat pads as a morpho‐functional unit,^[^ ^16^ ^]^ which was corroborated by our recent single‐cell atlas study.^[^ ^17^ ^]^ Our team has also reported that both MRI‐detected effusion synovitis^[^ ^18^ ^]^ and Hoffa's synovitis^[^ ^19^ ^]^ were strongly associated with KOA progression. Nevertheless, previous population‐based studies of GMB identified joint structural changes by radiography^[^ ^8^ ^]^ or ultrasound^[^ ^20^ ^]^ examinations, which could not identify effusion and/or Hoffa's synovitis. For magnetic resonance imaging (MRI), it has been suggested to use for screening synovitis (including both effusion and Hoffa's synovitis) in OA clinical trials by experts.^[^ ^2^, ^14^ ^]^

**Figure 1:**
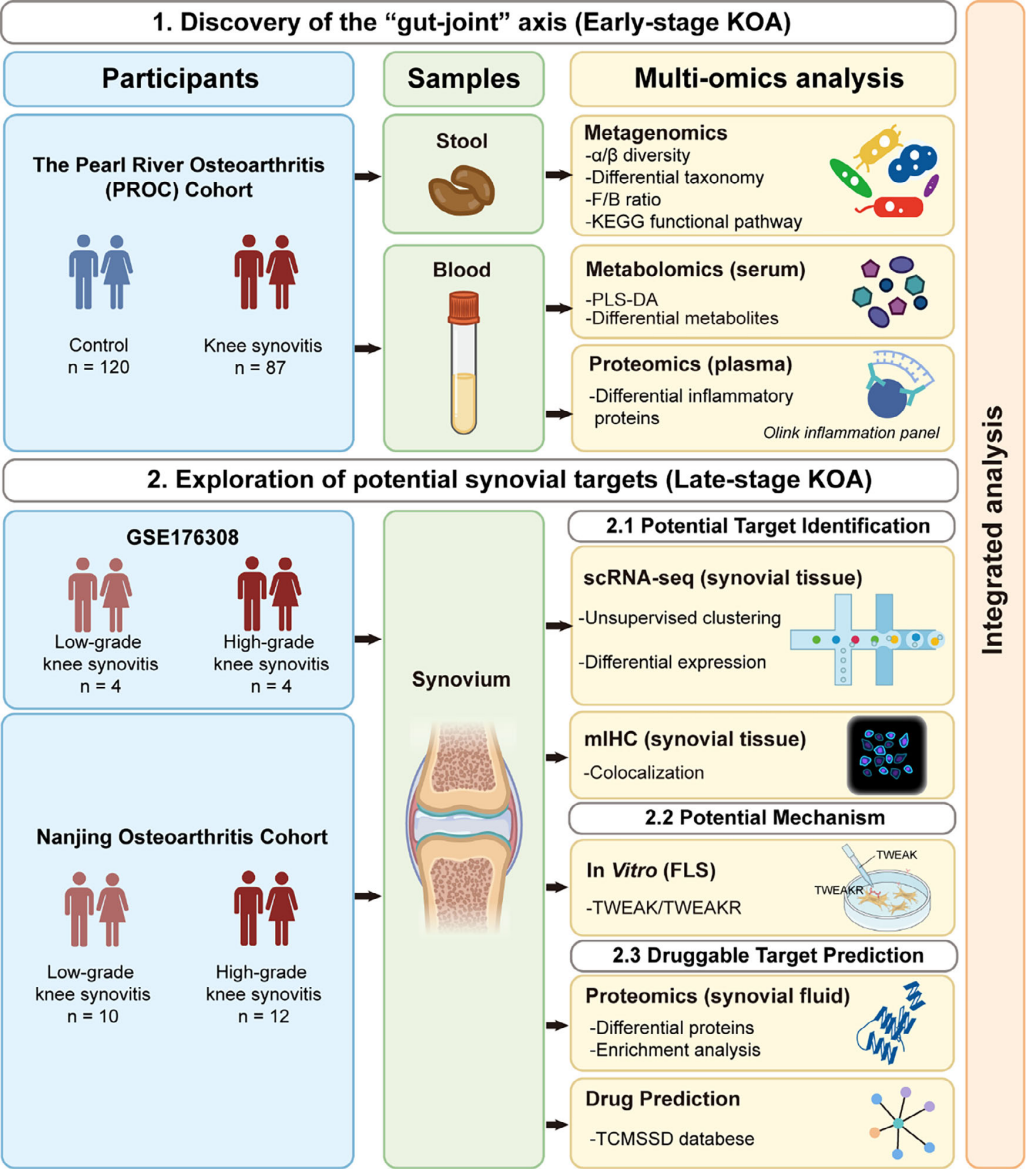
Overview of the study design and participants. This study integrates multi‐omics studies of fecal metagenomics, circulating metabolome and proteome in the Pearl River Osteoarthritis Cohort (PROC), in which knee synovitis was detected using MRI, and synovial proteomics, histological analysis, and in vitro study in the Nanjing Osteoarthritis Cohort (NOC), as well as a public single‐cell RNA sequencing dataset of synovial fibroblasts.

Overall, further population‐based multi‐omics studies of MRI‐detected knee synovitis and more direct evidence on synovium are urgently warranted to elucidate potential “gut‐joint axis” and to explore potential targets for knee synovitis. Here, we integrated multi‐omics studies of fecal metagenomics and circulating metabolomics and proteomics in middle‐aged and elderly subjects from the Pearl River Osteoarthritis Cohort (PROC), an observational study in Southern China, with detection of knee synovitis using MRI. Then, the synovial proteomics, histological and cellular studies in the Nanjing Osteoarthritis Cohort (NOC), as well as single‐cell study provided further direct evidence on potential mechanism (**Figure** **1**). Our study has generated a novel “gut‐joint axis” in knee synovitis and may provide novel targets for synovitis treatment.

## 2 Results

### 2.1 Study Population Characteristics

The flowchart depicts the selection process (Figure S1A, Supporting Information). The current study included 207 participants, including 87 with knee synovitis and 120 without. The baseline characteristics are presented in **Table** **1**.

**Table 1:** Clinical characteristics of included participants of PROC study at baseline.^a)^

Characteristic	Healthy Control (*N* = 120)	Synovitis (*N* = 87)	*P* value
Age (years)	58.0 (52.0, 64.0)	65.0 (58.5, 68.0)	< 0.0001
Female, n (%)	89 (74.2)	69 (79.3)	0.49
BMI (kg/m2)	23.6 (21.9, 25.6)	25.2 (22.6, 27.1)	0.0013
K‐L Grade, n (%)			< 0.0001^b)^
0	24 (20.0)	4 (4.6)	
1	71 (59.2)	26 (29.9)	
2	17 (14.2)	27 (31.0)	
3	7 (5.8)	25 (28.7)	
4	1 (0.8)	5 (5.8)	
Knee Synovitis			‐
Effusion synovitis alone	‐	10 (11.5)	
Hoffa's synovitis alone	‐	53 (60.9)	
With both	‐	24 (27.6)	
Knee injury history, n (%)	3 (2.5)	7 (8.1)	0.099
WOMAC Total (range 0–2400)	148.0 (76.8, 251.0)	266.0 (127.5, 620.0)	< 0.0001
WOMAC pain (range 0–500)	45.5 (20.8, 70.0)	70.0 (34.0, 134.5)	0.0010
WOMAC stiffness (range 0–200)	4.0 (0.0, 23.3)	14.0 (0.0, 38.0)	0.0069
WOMAC function (range 0–1700)	85.5 (41.8, 161.0)	176.0 (80.5, 406.0)	< 0.0001
NSAIDs, n (%)	5 (4.2)	9 (10.3)	0.14
TC (mmol/L)	5.44 (4.84, 6.35)	5.57 (4.82, 6.16)	0.70
TG (mmol/L)	1.28 (0.86, 1.73)	1.45 (1.02, 1.90)	0.070
BG (mmol/L)	5.12 (4.84, 5.40)	5.22 (4.85, 5.76)	0.061
Smoking, n (%)			0.91^b)^
Current	10 (8.3)	6 (6.9)	
None or used	110 (91.7)	81 (93.1)	
Alcohol, n (%)			0.99^b)^
Current	26 (21.7)	19 (21.8)	
None or used	94 (78.3)	68 (78.2)	
Tea, n (%)			0.99^b)^
Usually	52 (43.3)	37 (42.5)	
None or seldom	68 (56.7)	50 (57.5)	

^a)^ Data are presented as median (interquartile range) or number (percent). BMI, body mass index; K‐L grade, Kellgren‐Lawrence grade; WOMAC, The Western Ontario and McMaster Universities (WOMAC) Osteoarthritis Index; NSAIDs, Nonsteroidal anti‐inflammatory drugs; TC, Total cholesterol; TG, Triglyceride; BG, Blood glucose;

^b)^ *P* values were determined by the chi‐square test, otherwise by Wilcoxon's test. *P* < 0.05 was considered significant.

The group with synovitis differed significantly in age, body mass index, Kellgren‐Lawrence grade, and WOMAC scores from the control group. However, the two groups did not differ by NSAIDs used, lifestyles (smoking and intake of alcohol or tea) and glycolipidosis (TC, total cholesterol; TG, triglyceride; and BG, blood glucose), which were all usually considered as potential confounders of dysbiosis in GMB or blood metabolism. We also found that the severity of knee synovitis (overall scores) was significantly associated with knee WOMAC pain (multivariable linear regression analysis, *P* < 0.0001), and this association remained significant after additional adjustments for possible confounders (Table S1, Supporting Information). Since the knee pain was accepted as the consequence of synovitis, we adjusted for age, body mass index, Kellgren‐Lawrence grade, as well as gender, which were considered independent risk factors of knee synovitis, in subsequent analyses.

Moreover, 10 cases (4.8%) were identified as having effusion synovitis alone, 53 (25.6%) cases were identified to have Hoffa's synovitis alone, while 24 cases (11.6%) were identified as having both Hoffa's and effusion synovitis.

### 2.2 Dysbiosis of Gut Microbiome in Knee Synovitis

**Figure 2:**
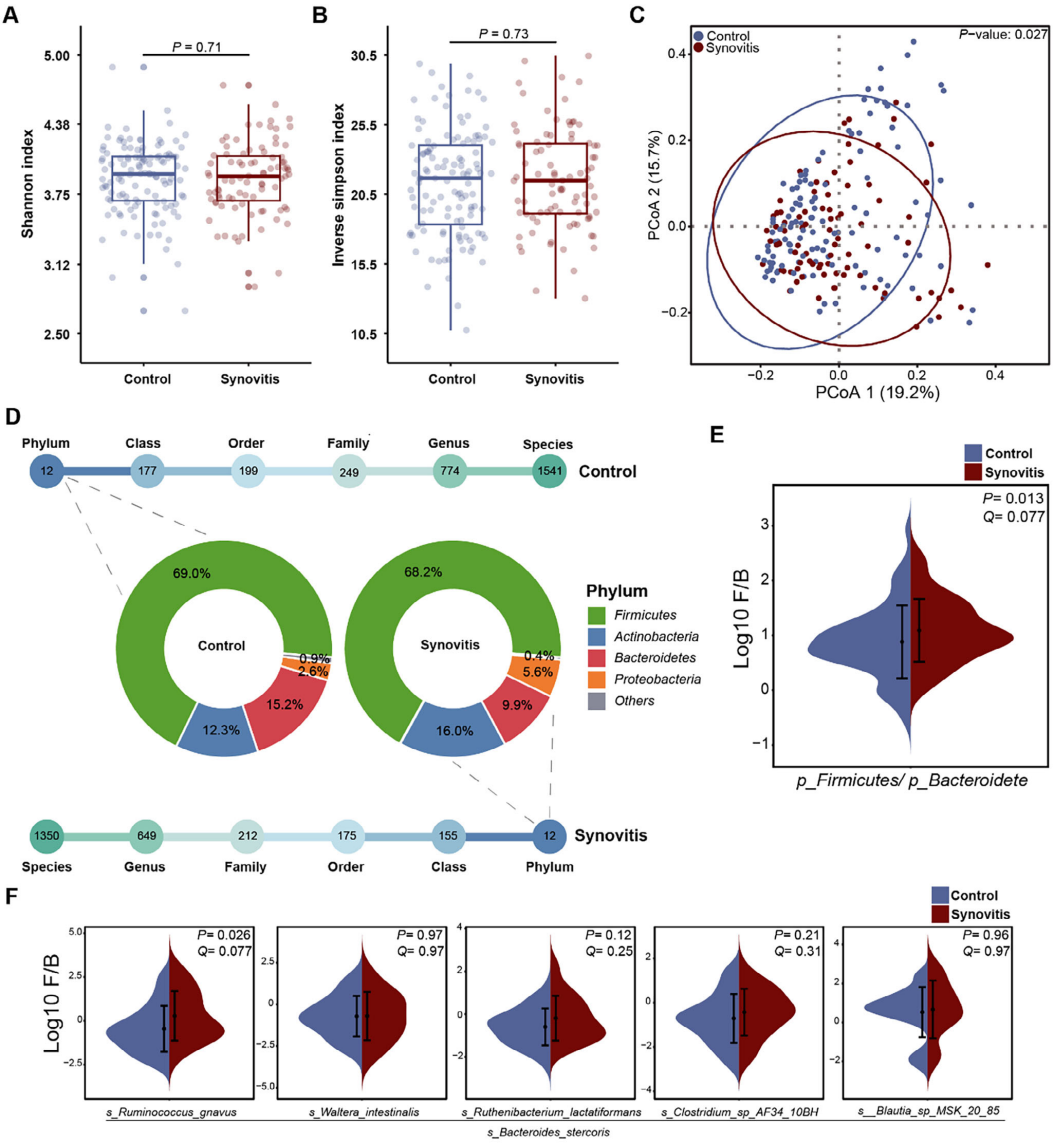
Gut microbiome profiles in individuals with and without knee synovitis. A,B) Box plots comparing the A) Shannon index and B) inverse Simpson index (alpha‐diversity) of participants without (control) or with (synovitis) knee synovitis. C) Principal Coordinates Analysis (PCoA) plot comparing the global composition of the synovitis group with that of the control, constructed by Bray‐Curtis distance (beta‐diversity). D) An overview of the number of unique taxonomies detected at each level, from phylum to species, for each group. The unknown and unclassified bacteria were classified to “others”. Donut plot showing the relative abundance (%) of different unique phyla. E) Split‐violin plot showing difference of F/B ratio at the phylum level between two groups. F) Split‐violin plots showing the difference in F/B ratio at species level between two groups. For inter‐group comparisons of F/B ratio, only cases with log_10_ (F/B) < 3 were included. Wilcoxon rank sum test was performed. The difference that fulfilled both *P* value < 0.05 and Q value < 0.2 (Benjamini‐Hochberg adjustment for *P* value) is considered significant.

All the fecal samples were subjected to shotgun metagenomic sequencing to detect the alteration of the gut microbiome in knee synovitis, compared to the controls. Although gut microbial α‐diversity (i.e., Shannon index and inverse Simpson index) demonstrated no difference between groups (*P* = 0.71, **Figure** **2**A and *P* = 0.73, Figure 2B, respectively), the β‐diversity differed significantly between the two groups (*P* = 0.027, PERMANOVA, Figure 2C; Table S2, Supporting Information). In total, there were 2,922 and 2,552 single taxonomies in the control and group with synovitis, respectively. At the phylum level, the dominant phyla in controls were *Firmicutes* (69.0%), and *Bacteroidetes* (15.2%), whereas *Firmicutes* (68.2%) and *Actinobacteria* (16.0%) were the major compositions in synovitis group, followed by reduced *Bacteroidetes* (9.9%) (Figure 2D). Of note, there was a significant increase in *Firmicutes*/*Bacteroidetes* (F/B) ratio for the synovitis group compared with the control at the phylum level (*P* = 0.013, Q = 0.077, Figure 2E).

To gain more insights into which gut‐microbiome taxonomies drive the association with the presence of knee synovitis at a species level, we performed MaAsLin2 analysis by adjusting for covariates (age, sex, body mass index and Kellgren‐Lawrence grade), and found that the abundances of seven species were significantly associated with the presence of knee synovitis (Figure S2A and Table S3, Supporting Information). These taxa were identified as synovitis‐related features. Of these, *Bacteroides stercoris* was significantly depleted not only at phylum and species levels, but also at class, order, family and genus levels, respectively (Figure S2B–G, Supporting Information). These findings strongly suggested a potential dysbiosis characterized by an altered Firmicutes/Bacteroidetes (F/B) ratio, which had been frequently associated with inflammatory diseases such as rheumatoid arthritis (RA) and inflammatory bowel disease (IBD). It was observed that, except for the species level, no significant difference of *Ruminococcus gnavus* between synovitis and control groups at diverse taxonomic levels (Figure S2H–M, Supporting Information), strongly indicated that differences in the Firmicutes/Bacteroidetes (F/B) ratio in the synovitis might be mainly driven by the declines of *Bacteroidetes* (*Bacteroidetes stercoris*) at various taxonomic levels (Figure S2B–G, Supporting Information). Furthermore, the F/B ratio between synovitis‐related species classified to *Firmicutes*, and the species classified to *Bacteroides stercoris* was calculated, respectively (Figure 2F). The ratio of *Ruminococcus gnavus*/*Bacteroides stercoris* significantly increased in the synovitis group (*P* = 0.026, Q = 0.077). The elevated F/B ratio at the species level might be attributed to the decline of *Bacteroidetes stercoris* and/or the increase of *Ruminococcus gnavus*.

### 2.3 Alteration in Serum Metabolites and Their Links to Gut Microbial Function in Knee Synovitis

**Figure 3:**
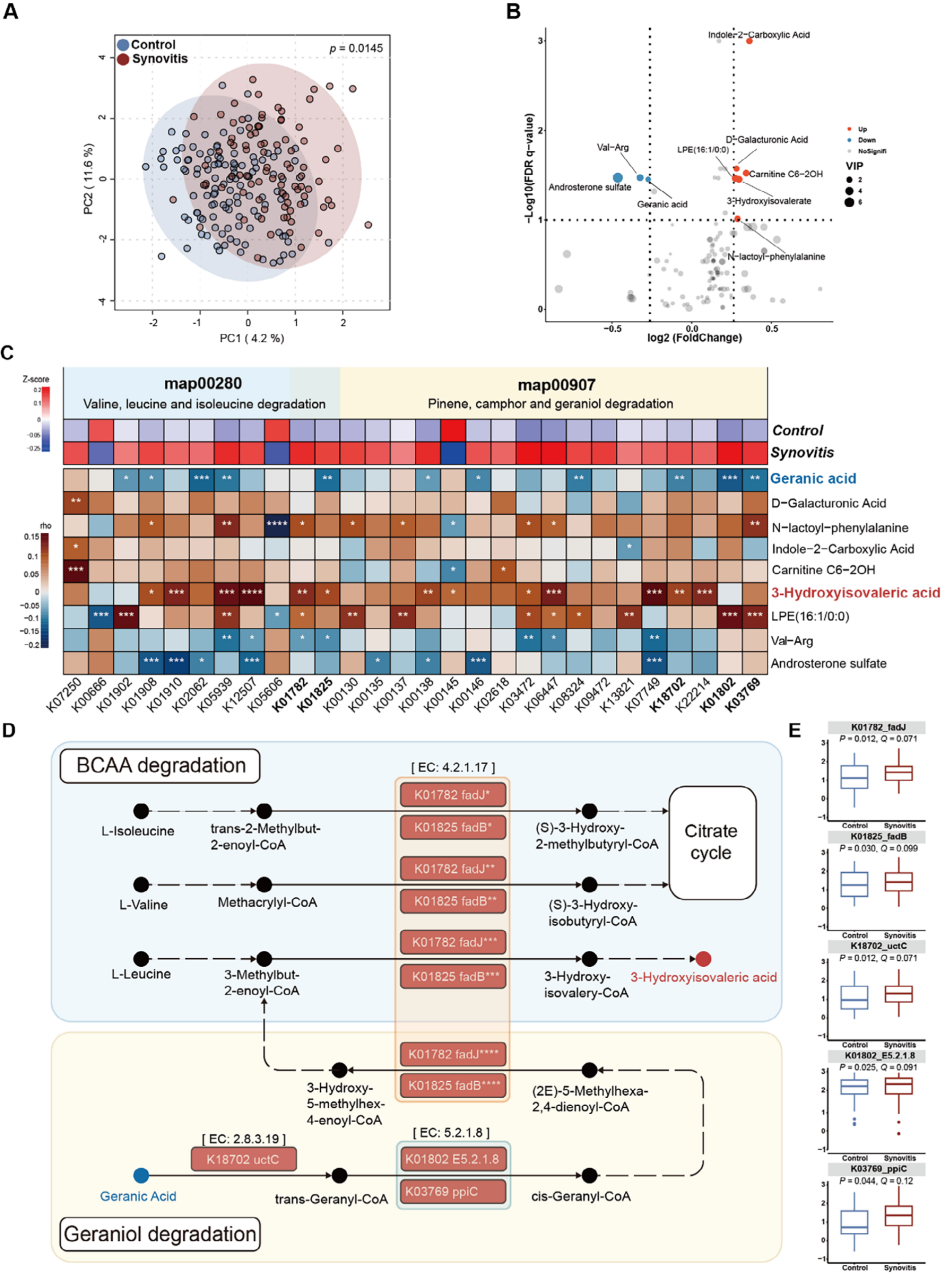
Synovitis‐related alterations in host serum metabolome and their links to gut microbial function. A) Partial least‐squares‐discriminant analysis (PLS‐DA) for alterations of metabolites in the synovitis and control groups. B) Volcano plot of the significantly different metabolites between the control and knee synovitis groups. Fold change cutoff is > 1.2 or < 0.83 (up, red dots; down, blue dots; not significant, grey dots), False discovery rate (FDR)‐adjusted *P* value < 0.2 was set as threshold. C) Heatmap displays the differential expression of KEGG orthologs (KOs) in synovitis and control groups (upper), and correlation between KOs and synovitis‐associated serum metabolites (lower), within two pathway modules modified from KEGG pathway maps “Valine, leucine and isoleucine degradation” and “Pinene, camphor and geraniol degradation”, respectively (partial Spearman, adjusted for age, gender, body mass index and Kellgren‐Lawrence grade, ^*^Q < 0.2, ^**^Q< 0.1, ^***^ Q < 0.05, ^****^Q < 0.01; Q, adjusted P value). D) KO genes, represented as boxes and highlighted in red for elevation in the synovitis group, are shown in two metabolic pathway modules, while red indicates upregulated metabolites in synovitis, and blue indicates the opposite. The pathways were generated on the basis of KEGG pathway maps. E) The box plots showing the log_10_ (relative abundances) of differential KOs between the two groups (Wilcoxon rank sum test with Benjamini‐Hochberg correction, fulfilling both *P* < 0.05 and Q < 0.2 is considered statistically significant). Asterisks represent different reactions in K01782 fadJ and K01825 fadB according to KEGG: ^*^ for R04204, ^**^ for R04224; ^***^ for R04137; ^****^ for R08093.

The serum metabolomics reflects the metabolic changes in the host.^[^ ^21^ ^]^ Thus, we conducted metabolic profiling in all serum samples, and a total of 703 metabolites were identified. After quality control, 527 metabolites remained for further analysis. The Partial Least Squares Discriminant Analysis (PLS‐DA) model was established to detect the disparities in serum metabolites between groups, and its prediction accuracy was validated by a permutation test (permutation test, *p* = 0.016; Figure S3, Supporting Information). A total of 111 candidate metabolites were obtained with the value of Variable Importance in the Projection (VIP) > 1 that separated synovitis from controls (**Figure** **3**A; Table S4, Supporting Information). Then, six metabolites were found to be significantly elevated in synovitis, including LPE (16:1/0:0, HMDB0011474), 3‐hydroxyisovaleric acid (HMDB0000754), Carnitine C6‐2OH (HMDB0000552), indole‐2‐carboxylic acid (HMDB0002285), N‐lactoyl‐phenylalanine (HMDB0062175), and D‐galacturonic acid (HMDB0002545). On the other hand, three metabolites were significantly decreased, including androsterone sulfate (HMDB0002759), Val‐Arg (HMDB0029121), and geranic acid (HMDB0036103). These metabolites were also identified as synovitis‐related features (Figure 3B).

To examine the links between synovitis‐related metabolites and microbial function, we first identified a total of 1,760 differential Kyoto Encyclopedia of Genes and Genomes Orthology (KOs) in metagenomics differing between the two groups (Table S5, Supporting Information). Furthermore, the synovitis was found to be mainly characterized by disturbed metabolism of 3‐hydroxyisovaleric acid (map00280, valine, leucine and isoleucine degradation, i.e., branched‐chain amino acid degradation/BCAA degradation) and geranic acid (map00907, pinene, camphor and geraniol degradation) (Figure 3C; Table S6, Supporting Information). More importantly, we explored the potential microbial genes in modulating hematological metabolism by mapping the corresponding KOs (according to enzyme commission numbers in KEGG pathway) to disturb the “BCAA degradation” and “Geraniol degradation.” Here, KOs including K01782 *fadJ*, and K01825 *fadB* and the relevant metabolite of 3‐hydroxyisovaleric acid in serum, were found to be enriched simultaneously in synovitis (Figure 3D,E). It suggested that excessive 3‐hydroxyisovaleric acid was closely related to over‐activated KOs in GMB. The similar findings were observed in K18702 *uctC*, K01802 *E5.2.1.8* and K03769 *ppiC*, whereas geranic acid was decreased (Figure 3D,E). These findings support that disrupted microbial genes may lead to over‐degradation of geranic acid, and subsequently induce excessive downstream 3‐hydroxyisovaleric acid in the host.

Moreover, the potential links between D‐galacturonic acid, which was enriched in synovitis, and differing KOs were also displayed (map00520, amino sugar and nucleotide sugar metabolism, Figure S4, Supporting Information).

### 2.4 Changes of Inflammatory Proteins in Plasma and Their Links to Microbial Function in Knee Synovitis

Plasma proinflammatory proteins can uncover the inflammatory response in the host.^[^ ^22^ ^]^ The Olink Explore platform employs a highly sensitive detection method based on high‐throughput proteomics.^[^ ^23^ ^]^ Thus, the plasma levels of 92 inflammatory proteins were measured using the Olink Inflammation Panel. After quality control, 75 candidate proteins remained and were analyzed (Table S7, Supporting Information). The associations between expression levels of candidate proteins and the presence of synovitis were analyzed. Fibroblast growth factor 23 (FGF‐23) and Interleukin 17C (IL‐17C) were found to be positively correlated with the presence of synovitis (Figure S5A, Supporting Information). The tumor necrosis factor (Ligand) superfamily, member 12 (TWEAK) was found to be negatively correlated with the presence of synovitis. The associations remained unchanged after sensitivity analysis with further adjustments (Table S8, Supporting Information).

Furthermore, the links between FGF‐23, IL‐17C or TWEAK and differing KOs of branched‐chain amino acid degradation (map00280) or pinene, camphor and geraniol degradation (map00907) were analyzed (Figure S5B and Table S9, Supporting Information). The FGF‐23 was positively correlated with K01802, which is involved in the geraniol degradation pathway, suggesting that dysbiosis of GMB in geraniol metabolism might induce FGF‐23 in host. The TWEAK was found to be negatively correlated with K01825, which was involved in both BCAA and geraniol metabolism, which indicating that disturbed BCAA and geraniol metabolism might have contributed to the reduction of TWEAK in the host.

### 2.5 Multi‐Omics Detections of Synovium and Synovial Fluid in the Nanjing Osteoarthritis Cohort

**Figure 4:**
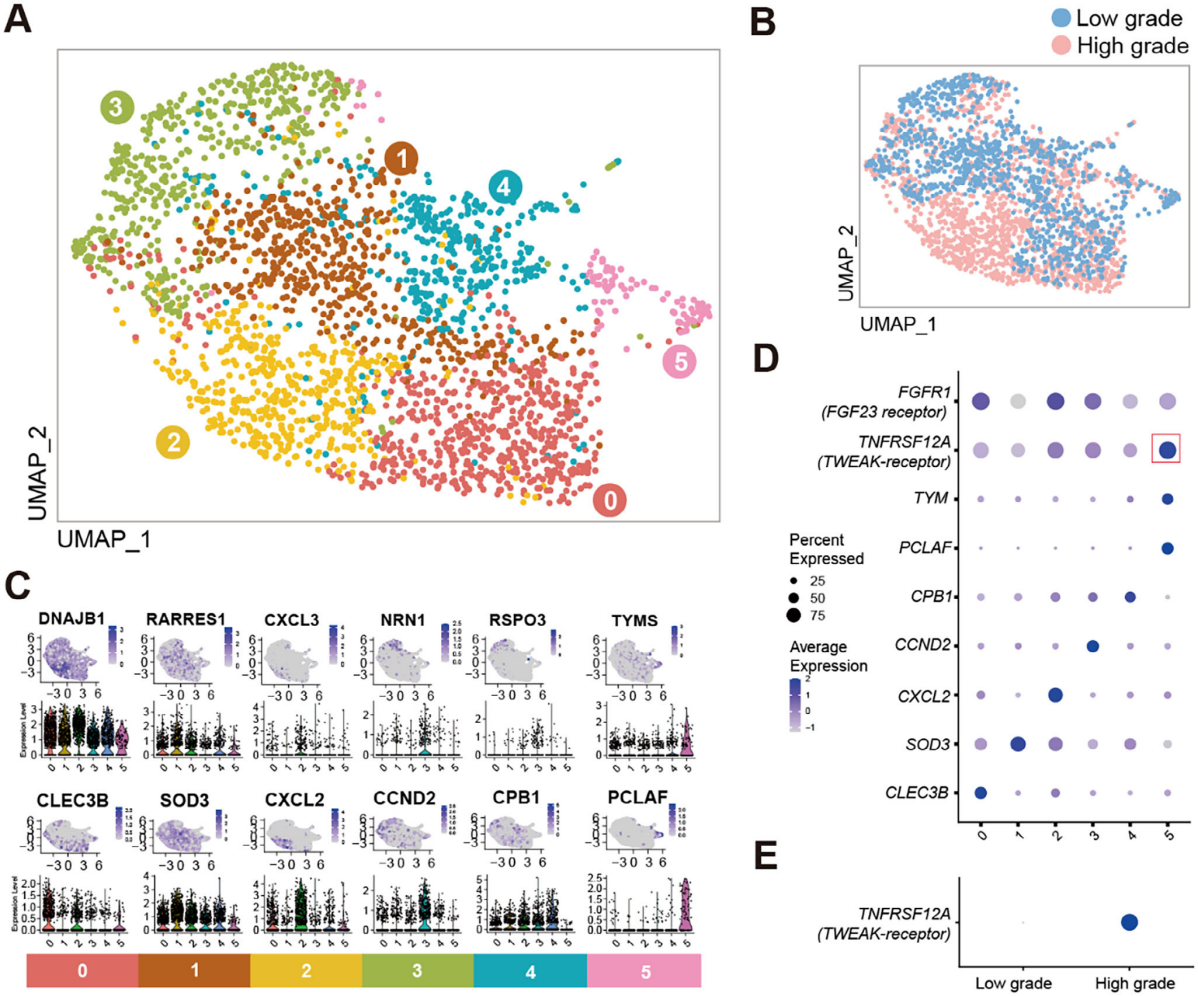
Single cell RNA‐sequencing identifies TWEAK receptor in synovium from OA patients with synovitis. A,B) The Uniform Manifold Approximation and Projection (UMAP) plots exhibit (A) the landscape and (B) the sample origin of synovial fibroblasts, from low‐grade synovitis (*N* = 4) and high‐grade synovitis (*N* = 4), with identification of 6 fibroblast subsets (cluster 0–5). C) Feature plots depicting expression of specific markers for each cluster on the UMAP map, with violin plots below displaying the expression levels of these markers (y‐axis) across the distinct clusters (x‐axis). D,E) Dotplots showing D) expression level of specific marker genes of distinct clusters and receptors for TWEAK and FGF23, respectively, and E) group difference of receptors for TWEAK.

To detect the expression of the receptors for TWEAK (TWEAKR, TNFRSF12A) and FGF‐23 (FGFR1) in synovium, we collected a publicly accessible single‐cell RNA‐sequencing dataset (GSE176308) of human synovial fibroblast, selecting cells from early‐stage OA patients with low‐grade synovitis (n = 4) and end‐stage OA patients with high‐grade synovitis (*N* = 4) to conduct re‐analysis. A total of 2,518 cells (low‐grade, 1,115 cells; high‐grade, 1,403 cells) in 6 clusters were identified by unbiased clustering (**Figure** **4**A,B). The specific marker genes of each cluster were also displayed (Figure 4C). Of note, the TWEAKR was highly expressed in cluster 5, which was characterized by genes of PCLAF and TYM, while the former one was newly identified by reanalysis (Figure 4D). Furthermore, TWEAK receptor (TNFRSF12A) was observed to be highly expressed in cells of high‐grade synovitis (Figure 4E).

**Figure 5:**
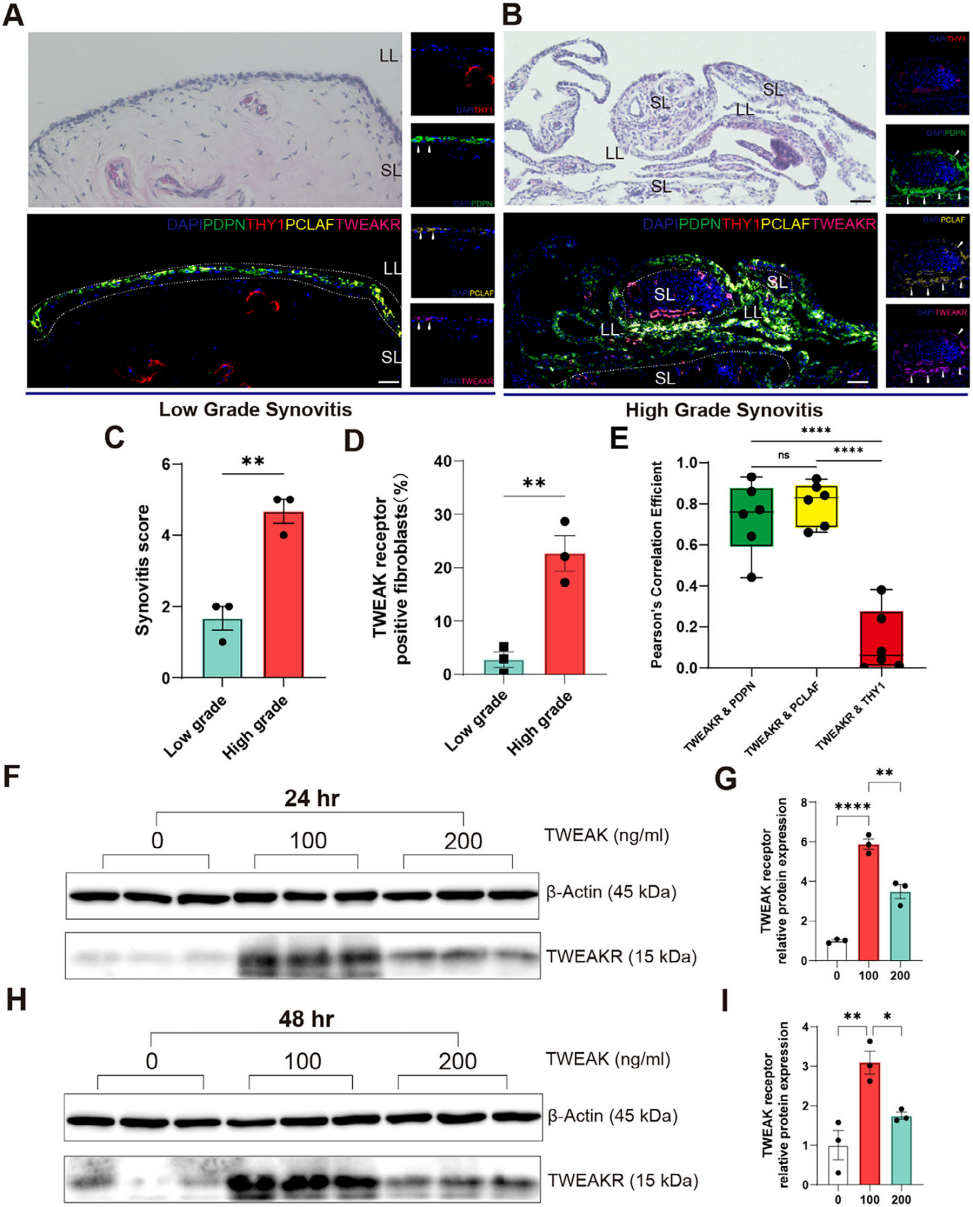
Expression and regulation of TWEAK receptor in synovium of knee OA patients from Nanjing Osteoarthritis Cohort (NOC) study. A,B) Representative HE and mIHC staining of human synovial tissues with (A) low‐grade and (B) high‐grade synovitis from the NOC, stained for anti‐THY1 (CD90, red), anti‐PDPN (green), anti‐PCLAF (yellow) and TWEAKR (TNFRSF12A, pink); white arrows show THY1+PDPN+TWEAKR+ cells; scale bar, 50 µm. C) Synovitis score quantification of human synovial tissues with low‐grade and high‐grade synovitis (*N* = 3); D) Colocalization of TWEAKR and PDPN/PCLAF/THY1. Data is presented and compared with each other, as Pearson's correlation coefficient. E) Quantification and difference of human synovium with TWEAKR+ fibroblasts between the tissues with low‐grade synovitis and high‐grade synovitis. Data presented as mean ± SEM or median (interquartile range). Unpaired t‐test or one‐way ANOVA. ^*^ *P* < 0.05, ^**^ *P* < 0.01, ^***^ *P* < 0.001, ^****^ *P* < 0.0001.

To further verify the increased expression of TWEAKR in the synovium with high‐grade synovitis, the knee joint synovial tissues and synovial fluid were collected from the Nanjing Osteoarthritis Cohort (NOC, Table S10, Supporting Information). First, haematoxylin and eosin (H&E) staining and multiplex immunohistochemistry (mIHC) were performed for the synovium (**Figure** **5**A,B; Figure S6A–D, Supporting Information). The samples with high‐grade synovitis were examined by histology with significantly higher synovitis scores (Figure 5C). The TWEAKR was mainly expressed in samples with high‐grade synovitis (Figure 5D), and colocalized with PCLAF (cluster 5) and PDPN (lining layer), instead of THY1 (sub‐lining layer) (Figure 5E; Figure S6, Supporting Information). These findings suggested that the synovium with high‐grade synovitis expressed higher TWEAK receptors in the lining layers, especially in PCLAF+ FLS, than those with low‐grade synovitis.

Subsequently, human fibroblast‐like synoviocytes (FLS) were isolated from synovial samples of knee OA patients in the NOC and cultured. The FLS cells were treated with human recombinant TWEAK protein with ascending concentrations for 2 or 3 days (Figure 5F–I). The results revealed that as the concentration of TWEAK increased from 100 to 200 ng mL^−1^, the expression level of TWEAKR was reversely downregulated. It suggests that TWEAK downregulation in synovitis may conversely enhance synovial TWEAKR activation, contrasting with the upregulated TWEAK observed in the control group.

To further understand the biology of TWEAKR in synovium, and to determine its potential role in synovitis, the correlation analysis between the proteins from synovial fluid and grades of synovitis (Table S11, Supporting Information) and functional analyses (Gene Ontology) were utilized. We found that high‐grade synovitis was closely associated with enhanced immune response and impaired ability of antioxidants (Figure S7A, Supporting Information). In addition, the STRING protein‐protein interaction network analysis of TWEAKR found a sum of 6 proteins with a co‐expressed pattern (Table S12, Supporting Information) were significantly and positively correlated with high‐grade synovitis in synovial fluid (Figure S7B, Supporting Information).

Finally, using the TCMSSD database (http://tcmssd.ratcm.cn), we predicted ingredients with therapeutic potential for TWEAKR (TNFRSF12A) and its co‐expressed proteins (except for CARHSP1, no predicted ingredients shared with TWEAKR) as synovitis targets. At TCMSSD, the ingredients were all extracted from Chinese traditional drugs, and candidate ingredients were screened depending on reported associations with the aforementioned proteins.^[^ ^24^ ^]^ The 17‐beta‐estradiol (*N* = 6) was identified as the ingredient with the highest potential (Figure S7C, Supporting Information), followed by quercetin (*N* = 5) and 3,4‐benzopyrene (*N* = 4).

### 2.6 Establishment of Biological Network and Machine Learning Models by Multi‐Omics Features

**Figure 6:**
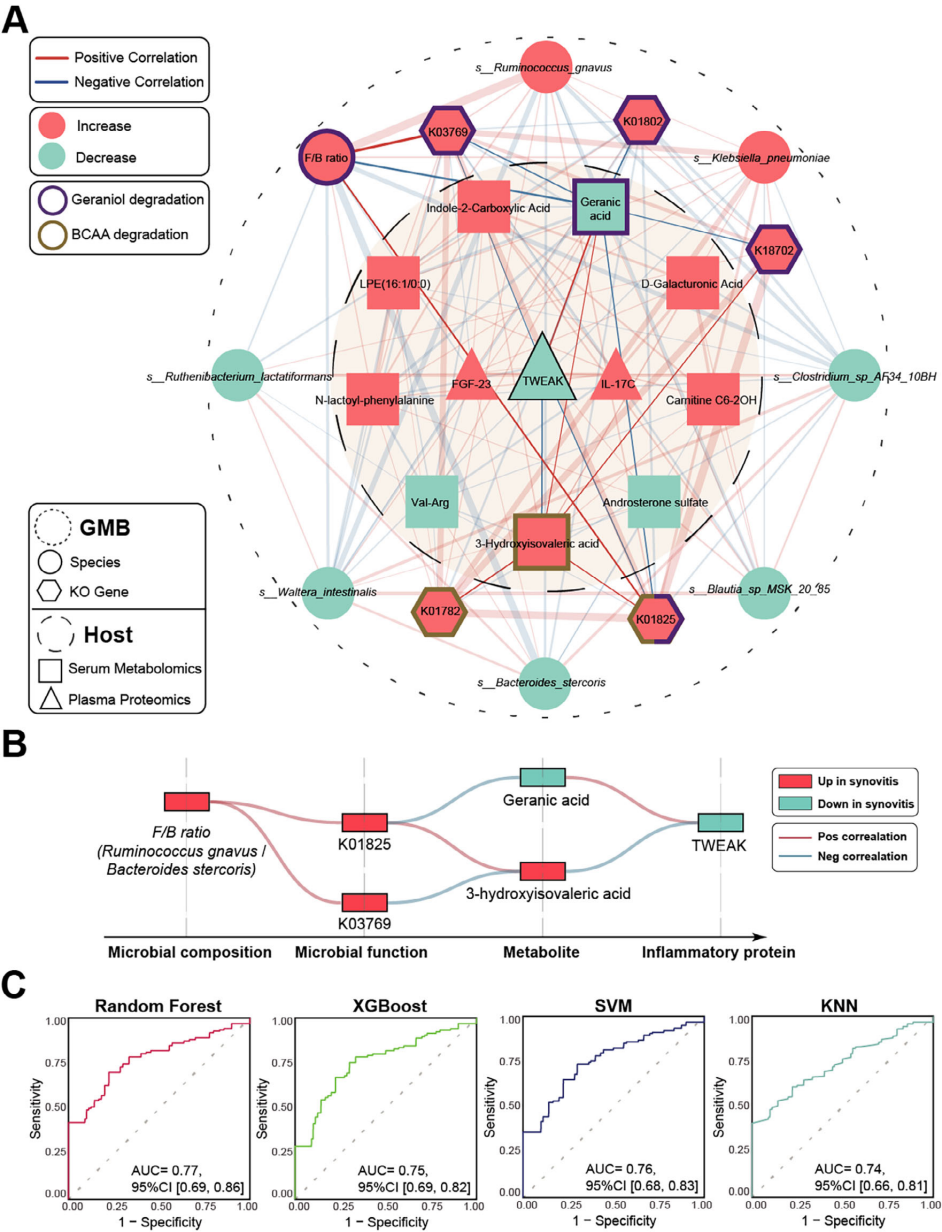
Integrated network and machine learning models of multi‐omics features in knee synovitis. A) Integrated network between species, KO genes, metabolites and inflammatory proteins. Only significant (Q < 0.2) links are shown. The colors of red and blue indicate the increased and decreased relative abundances of variables in synovitis relative to the control, respectively. Edges between nodes indicate partial Spearman's positive (light red) or negative (light blue) correlation, adjusting for sex, age, body mass index (BMI) and Kellgren‐Lawrence grade; edge thickness indicates the range of correlation coefficient. F/B ratio represents the ratio of relative abundance for *Ruminococcus gnavus (Firmicutes)/Bacteroides stercoris (Bacteroidetes)*. B) The key “microbiome‐metabolite‐immunity” interaction links were exhibited. Correlation analyses were performed by partial Spearman. Links between F/B ratio and KOs were limited in cases with log_10_ (F/B) < 3. C) The Receiver operating characteristic curves of random forest, XGBoost, support vector machine (SVM) and k‐nearest neighbors (KNN) models were performed to evaluate the predictive performances for the presence of synovitis by multi‐omics features, respectively.

To dissect the interactions between the GMB and host immunometabolism that might underlie synovitis, we performed correlation analysis between differentially abundant species, KO genes, metabolites, and inflammatory proteins, and generated network diagrams with representative signatures (**Figure** **6**A). The key findings exhibited that the increased F/B ratio in GMB was closely associated with disturbed leucine and geraniol metabolism, and following the immune response of TWEAK in the host (Figure 6B). However, a significant correlation between the F/B ratio and D‐galacturonic acid had not been observed.

Last, we attempted to establish a non‐invasive predictive model using synovitis‐related features, including clinical characteristics (WOMAC pain), GMB (*Ruminococcus gnavus* and *Bacteroides stercoris*), microbial function (K01825, K03769), metabolomics (geranic acid and 3‐HIA), and proteomics (TWEAK, IL‐17C and FGF‐23) in PROC. The predictive models were constructed, and nested cross‐validation was employed to ensure an unbiased assessment of the model performance (Figure 6C; Figure S8, Supporting Information). Using four algorithms, including random forest (mean AUC = 0.77), XGBoost (mean AUC = 0.75), SVM (mean AUC = 0.76), and k‐nearest neighbors (mean AUC = 0.74), all multi‐omics models exhibited better performances in predicting knee synovitis than each individual‐omics model. These findings further underscored the importance on candidate synovitis‐related features.

**Figure 7:**
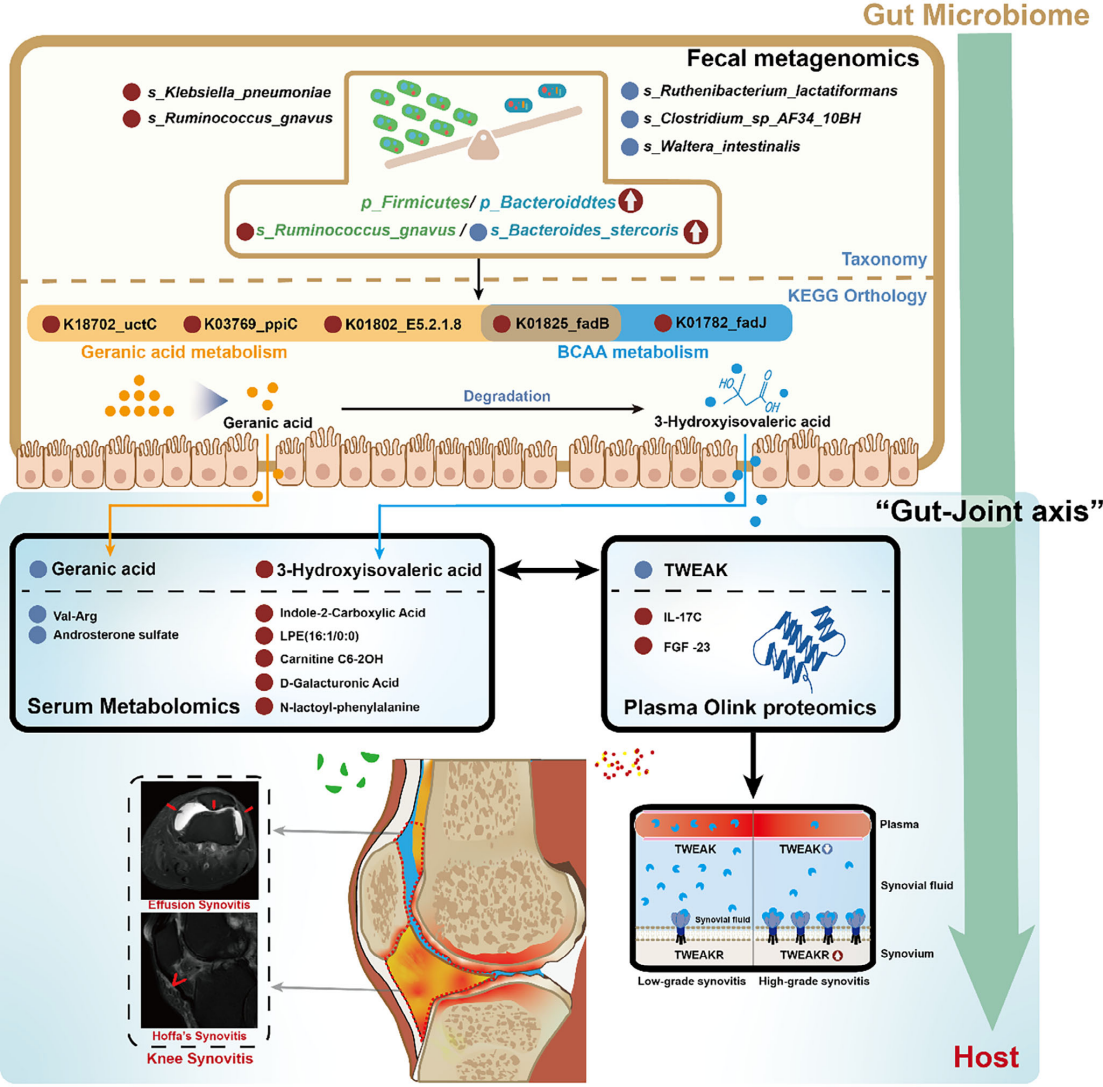
Schematic diagram of “Gut‐Joint Axis” in knee synovitis. The key multi‐omics features of “gut‐joint axis" in knee synovitis were presented. F/B ratio, Firmicutes/Bacteroidetes ratio; S, species; KO, KEGG Orthology.

The synovitis‐related multi‐omics characterizations and underlying mechanism of “gut‐joint axis” in knee synovitis were also summarized (**Figure** **7**).

## 3 Discussion

Although previous studies have demonstrated the alterations of the gut microbiome or circulating metabolites in synovitis,^[^ ^11^, ^20^ ^]^ there are still unresolved issues, particularly the underlying mechanism of the interactions between “gut microbiota” and “host immune‐metabolism.” To the best of our knowledge, this is the first study to decipher the underlying pathological mechanism of knee synovitis through “gut‐joint axis” by integrating metagenomic, metabolomic, proteomic, single‐cell study data and mIHC staining, as well as identifying both Hoffa's and effusion synovitis using MRI. The major findings were that increased F/B ratio in GMB was closely associated with disturbed leucine and geraniol metabolisms, which were subsequently correlated with reduced TWEAK in circulation and expanded TWEAK receptor in synovium. The relevant multi‐omics features were successfully utilized to develop non‐invasive predictive models by different machine learning algorithms.

Conventional imaging modalities, such as radiography or ultrasound, have shortcomings in the detection of synovitis, particularly in the case of Hoffa's synovitis. X‐ray imaging is not able to visualize the synovial pathogenesis. Ultrasonography, despite its low cost and convenience, is heavily reliant on the practitioner's skills and experience, while was rarely applied in Hoffa's synovitis identification.^[^ ^25^ ^]^ Thus, MRI is accepted as a preferred approach for structural OA assessment.^[^ ^6^, ^14^ ^]^ In current study, a significant association between the severity of knee synovitis (overall score of Hoffa's and effusion synovitis) and knee pain were observed, indicating the close relationship between MRI‐detected synovitis and knee symptoms. More importantly, a large proportion (60.9%, Hoffa's synovitis/knee synovitis) of cases detected by the MRI approach might have been misdiagnosed or underdiagnosed by the conventional strategy of identifying effusion synovitis only. A recent trial of MRI‐detected effusion‐synovitis did not achieve a significant effect of a pain reliever.^[^ ^26^ ^]^ It might result from an underestimation of the cases without effusion synovitis but with Hoffa's synovitis. Hence, the application of MRI is a prominent strength of our study.

In terms of the alteration of microbiome composition, the species of *Streptococcus* was observed to be positively associated with knee joint effusion in the Rotterdam study,^[^ ^27^ ^]^ while the fungal genus of *Schizophyllum* was found inversely correlated with effusion synovitis in Xiangya osteoarthritis study.^[^ ^20^ ^]^ However, we for the first time revealed the elevation of F/B ratio in knee synovitis, especially enriched *Ruminococcus gnavus* (species of *Firmicutes*) and depleted *Bacteroides stercoris* (species of *Bacteroidetes*). The disparate findings across each study might be attributed to regional variations and be more likely to the selection of populations using different diagnostic modalities for synovitis. Of note, the increasing F/B ratio is widely accepted as a risk factor of inflammatory diseases,^[^ ^28^ ^]^ and suppressing the abnormal elevation of the F/B ratio could delay inflammatory progression.^[^ ^29^ ^]^ Thus, we proposed that rebalancing F/B ratio might be an effective way to relieve knee synovitis and pain.

The microbiome‐related alterations of metabolites and relevant proinflammatory cytokines were also reported to be associated with osteoarthritis,^[^ ^13^ ^]^ but the underlying mechanism in knee synovitis is still uncovered. We herein, for the first time, identified alterations of 3‐HIA and geranic acid as critical changes for knee synovitis in serum. The geranic acid, namely 3,7‐dimethyl‐2,6‐octadienoic acid, is not only a polyunsaturated fatty acid, but also a double bond isomer of nerolic acid.^[^ ^30^ ^]^ It is widely known for its anti‐bacterial and anti‐inflammatory capacity.^[^ ^31^ ^]^ In the current study, geranic acid was found to be significantly decreased in the serum samples of subjects with synovitis. Moreover, this process was not isolated. The products of geranic acid might be transferred to the metabolism pathway of 3‐hydroxyisovaleric acid (3‐HIA) though activating KOs in the synovitis group. The 3‐HIA, also known as 3‐Hydroxy‐3‐MethylButyric Acid, was found to be increased in serum samples of synovitis group. 3‐HIA is a natural secondary metabolite of the leucine pathway. When metabolic disorder‐induced high levels of 3‐HIA persists, it can act as an acidogen or a metabotoxin.^[^ ^32^ ^]^ It is also known as a biomarker for inflammatory disease.^[^ ^33^ ^]^ In addition, increased F/B ratio was positively correlated with geranic acid‐related KOs and 3‐HIA‐related KOs, respectively. Therefore, we proposed that dysbiosis of GMB might induce over‐degradation of geranic acid, and subsequently contribute to the lack of leucine and excessive generation of 3‐HIA in the host. Thus, supplementation of geranic acid or leucine might fill these metabolic deficits to delay the progression of knee synovitis.

Among the inflammatory cytokines altered in synovitis, TWEAK, also known as tumor necrosis factor ligand superfamily member 12 (TNFSF12), was found to be negatively associated with the presence of knee synovitis. It was also positively and negatively associated with geranic acid (decreased in synovitis) and 3‐HIA (enriched in synovitis), respectively. TWEAK was identified as decreased in several disorders, such as hepatic steatosis,^[^ ^34^ ^]^ and dementia.^[^ ^35^ ^]^ Being a kind of multi‐functional cytokine,^[^ ^36^ ^]^ the role of TWEAK in joint disease is still controversial. The levels of TWEAK were also found relatively decreased as the OA progressed in the synovial fluid^[^ ^37^ ^]^; while the TWEAK receptor (TWEAKR) were found over‐expressed in rheumatoid arthritis (RA) synovium.^[^ ^38^ ^]^ The scRNA‐seq analysis and mIHC staining demonstrated the expanded TWEAKR in high‐grade synovitis compared with low‐grade synovitis in current study. We also found down‐regulation of TWEAKR by increasing human recombinant TWEAK protein, suggesting that lower TWEAK in synovitis might induce up‐regulated expression of TWEAKR. Accordingly, we proposed that TWEAKR might be amplified in synovium with higher grade. Taken together, TWEAK‐TWEAKR might play a central role of innate‐immune‐response inhibition in healthy or low‐grade conditions, while being activated by a lack of geranic acid or excessive 3‐HIA induced by microbiome‐related alterations in high‐grade condition, and subsequently contribute to the aseptic inflammation.

Our study still has several potential limitations: First, based on a cross‐sectional design, the results obtained cannot establish a causal relationship between the identified multi‐omics biomarkers and knee synovitis. We believe that future studies will benefit from following participants longitudinally. Second, the sample size was relatively limited, and participants were derived from a single country with the same ethnicity, limiting the generalizability of the findings to other populations. Larger prospective follow‐up studies involving more ethnic groups and geographic regions remain to be conducted. However, external cohorts were selected to provide more direct evidence of synovial tissues at the single‐cell level in current study. The non‐invasive predictive models using four machine learning approaches, with inner loops (LOOCV) and outer loops (10‐fold cross‐validation) and multi‐omics features were also established to validate the robustness and reproducibility. Third, we didn't utilize the contrast‐enhanced MRI, which was considered as gold standard for identifying synovitis on imaging, but non‐contrast‐enhanced MRI is also a valuable tool for the early detection of synovial inflammation, particularly in light of the ethical and practical considerations.^[^ ^6^ ^]^ Last, we have only evaluated sequencing profiles, histology study, and preliminary in‐vitro experiments, but more in vitro and in vivo studies are expected.

In summary, our study for the first time revealed the knee synovitis‐associated “gut‐joint axis,” characterized by an increased F/B ratio, disturbed leucine and geraniol metabolism in circulation, as well as reduced TWEAK, and upregulated TWEAKR in synovium. Our findings may provide deeper insights into the understanding of the “gut‐joint axis” and reveal potential targets for synovitis treatment.

## 4 Experimental Section

### Study Population

The Pearl River Osteoarthritis Cohort (PROC) Study is an observational study that aims to investigate the natural history, risk factors, distinct subtypes and disease burden for knee osteoarthritis in Guangzhou, Southern China. From 2020 to 2022, the PROC study has prospectively recruited around 450 adults aged between 45 and 79 years old who self‐reported knee pain in at least one knee. Participant enrollment is ongoing, and the ultimate goal is to recruit 5,000 participants, with follow‐up every two years. The study collected data via face‐to‐face interviews, clinical examinations, imaging and biospecimens.

The underlying pathogenic mechanism of MRI‐detected knee synovitis was explored by integrating multi‐omics data. The current analyses initially included the first 300 participants in the PROC study recruited from 2020 to 2022, The exclusion criteria are as follows: 1) incomplete clinical and radiographic information; 2) incomplete biospecimens (stool, serum, and plasma); 3) self‐reported bilateral knee pain; 4) history of rheumatoid arthritis, psoriatic arthritis, gout, ankylosing arthritis and other autoimmune disease; 5) self‐reported antibiotic use 1 month prior to stool sample collection; 6) history of inflammatory bowel disease, gastrointestinal tract surgery, cancer within six months before enrollment; 7) severe OA requiring arthroplasty within six months; 8) coexisting primary diseases such as liver, hematological, autoimmune diseases; tumor or psychiatric disorders; 9) inability to cooperate or unwillingness to provide samples; 10) significant changes in smoking, alcohol intake and dietary habits within six months before enrollment. Detailed screening processes are illustrated in Figure S1A (Supporting Information). Finally, a total of 207 subjects were included in the PROC study as discovery cases. In addition, a total of 22 patients who received total knee replacement (TKR) were selected from the Nanjing Osteoarthritis Cohort Study (NOCS) as validated cases. Consent was obtained from all participants of the two cohorts whose samples were collected for profiling.

### Knee Synovitis Assessment—MRI Acquisition of Knee Synovitis

Participants were positioned in the supine position with knees in full extension and scanned with a fast extremity coil (Philip 3.0T, Ingenia, Nederlands) within a week of the other examinations. The scan sequences were set to include the sagittal proton density weighted‐spectral attenuated inversion recovery for Hoffa's synovitis (repetition time (TR) 4522 ms; echo time (TE) 45 ms; field of view (FOV) 185 × 185 × 119 mm; matrix 296 × 240 mm; slice thickness of 2 mm with a gap of 1 mm between slices), and axial proton density weighted‐spectral attenuated inversion recovery for effusion synovitis (repetition time (TR) 2237 ms; echo time (TE) 45 ms; field of view (FOV) 170 × 170 × 107 mm; matrix 272 × 203 mm; slice thickness of 5 mm with a gap of 1 mm between slices).

### Knee Synovitis Assessment—Intra‐Reader Agreement

The MRIs were read by two experienced researchers without any knowledge of the participants’ clinical status or without knowledge of the research hypothesis. In detail, the weighted kappa of inter‐observer reliability was 0.89 (95% CI: 0.84, 0.94) and 0.91 (95% CI: 0.87, 0.95) for effusion and Hoffa's synovitis, respectively.

### Knee Synovitis Assessment—Assessment

According to the MRI Osteoarthritis Knee Score (MOAKS) method,^[^ ^15^ ^]^ Hoffa's synovitis was graded from 0–3 (0 = normal, 1 = mild, 2 = moderate, and 3 = severe) according to the hyperintensity on the sagittal intermediate‐weighted fat‐suppressed sequence. Effusion‐synovitis was graded from 0 to 3 (grade 0 = none, grade 1 = small, grade 2 = medium, and grade 3 = large), according to the degree of capsular distension. Grade 1 represented fluid existing continuously in the retropatellar space, Grade 2 represented slight convexity of the suprapatellar bursa, and Grade 3 represented evidence of capsular distension (Figure S1B–E, Supporting Information). A summary score of synovitis (range 0–6), was calculated as the sum of both Hoffa‐synovitis scores and effusion scores.^[^ ^15^, ^39^ ^]^ The prevalence of synovitis was defined as described previously^[^ ^6^, ^40^ ^]^: one or both of Hoffa's synovitis and effusion synovitis with a grade ≥ 2 in PROC study. In NOCS study, MRI images were assessed before TKR surgeries, and cases with scores ≥ 4 were considered as high‐grade synovitis, otherwise as low‐grade synovitis.

### Sample Collection

All blood samples were collected as soon as the participants were enrolled. The serum samples were clotted at room temperature for 30 min, centrifuged at 3000 × g for 10 min and stored at −80 °C at Clinical Biobank Centre of Zhujiang Hospital until use. The plasma samples were frozen at −80 °C with ethylenediaminetetraacetic acid directly. At the same visit as blood samples, all participants were required to provide a minimum of 3.0 grams of stool samples. On the spot or at home, the stool samples were immediately stored at −20 °C until they were transported on dry ice to the Biobank Centre, and frozen at −80 °C in plastic tubes immediately. Fecal samples were processed strictly according to the guidelines of International Human Microbiome Standards (IHMS). The samples of synovial fluid (SF) from human knees were obtained from the NOCS study. Before TKR surgeries, specimens were frozen at −80 °C within 2 h of extraction, and were centrifuged for 15 min at 1800 relative centrifugal force (rcf) before freezing.

### Fecal Metagenomics—Sample Testing

There were two main methods in quality control (QC) for DNA samples: 1) DNA degradation degree and potential contamination was monitored on 1% agarose gels; 2) DNA concentration was measured using Qubit dsDNA Assay Kit in Qubit 2.0 Flurometer (Life Technologies, CA, USA). The OD value should be between 1.8–2.0. All samples passed the QC.

### Fecal Metagenomics—Library Construction

After all samples passed QC, DNA contents above 1 µg were used to construct the library. A total amount of 1 µg DNA per sample was used as input material for the DNA sample preparations. Sequencing libraries were generated using NEBNext Ultra DNA Library Prep Kit for Illumina (NEB, USA) following manufacturer's recommendations and index codes were added to attribute sequences to each sample. Briefly, the DNA sample was fragmented by sonication to a size of 350 bp, then DNA fragments were end‐polished, A‐tailed, and ligated with the full‐length adaptor for Illumina sequencing with further PCR amplification. At last, PCR products were purified by AMPure XP system. The libraries were analyzed for size distribution by Agilent 2100 Bioanalyzer, and quantified using real‐time PCR.

### Fecal Metagenomics—Sequencing

The clustering of the index‐coded samples was performed on a cBot Cluster Generation System according to the manufacturer's instructions. After cluster generation, the library preparations were sequenced on an Illumina NovaSeq platform, and paired‐end reads were generated as the raw data.^[^ ^41^ ^]^

### Fecal Metagenomics—Metagenomic Profiling

Raw data of metagenomic sequencing was pre‐processed using KneadData (v0.12.0, http://huttenhower.sph.harvard.edu/kneaddata), which integrates FastQC (v0.11.9, quality testing, http://www.bioinformatics.babraham.ac.uk/projects/fastqc), Trimmomatic (v0.39, data filtering^[^ ^42^ ^]^), and Bowtie2 (v2.5.1, decontamination for host sequence^[^ ^43^ ^]^), respectively. Trimmomatic was carried out using the default values: “SLIDINGWINDOW: 4:20 MINLEN:70.” The minimum length was determined as 70% of the input read length, and GRCH38 was taken as the host reference genome. The MetaPhlAn 4.0 taxa with low abundance (with mean relative abundance across samples < 0.1%), and pathways of HUMAnN 3.0.0 (https://huttenhower.sph.harvard.edu/humann3/) with mean relative abundance across samples < 0.001% were filtered out prior to downstream analyses. The primary classes of statistical testing were used throughout this analysis, including omnibus tests and per‐feature tests. The former analysis assessed whether the whole microbial community structure was significantly different on the basis of synovitis status, whereas the latter one assessed this for each feature (e.g., taxon, KEGG orthology/KO). To identify specific taxa significantly associated with the presence of knee synovitis, MaAsLin2 (Microbiome Multivariable Association with Linear Model 2) v1.10.0 was applied to count trends in abundance data, adjusting for age, gender, body mass index and Kellgren‐Lawrence grade. More details for metagenomic analysis are presented in the Supporting Information.

### Serum Metabolomic Profiling

The ultra‐performance liquid chromatography/tandem mass spectrometry (UPLC‐MS/MS) Metabolomics was performed using collected serum samples. Details for sample preparation, data acquisition, processing and quality control (QC) were summarized in Supplementary Materials and Methods. Partial least squares‐discriminant analysis (PLS‐DA) was performed to rank the significance of different metabolites between synovitis and control groups based on variable importance in projection (VIP) scores, and the prediction accuracy was validated by a permutation test (2,000 times permutations, *p* < 0.05). The metabolites with VIP scores >1 indicated that they contributed significantly to the separation between groups.

### Plasma Proteomics for Inflammatory Biomarkers

The “Olink Target 96 Inflammation panel”, which enabled the measurement of 92 biomarkers for each sample, was used to measure distinct inflammation‐related proteins in plasma.^[^ ^44^ ^]^ The panels use proximity extension assay technology (PEA), and the detailed information on the reliability and stability of this technology was portrayed elsewhere.^[^ ^44^ ^]^ Normalized Protein Expression (NPX) values, which were on a log_2_ scale, were calculated to reflect protein levels after normalization at Olink's facilities.^[^ ^44^ ^]^ Quality control (QC) was performed as follows: biomarkers with > 25% of the NPX values below the minimum lower limit of detection (LOD) were excluded, and NPX values below the LOD were replaced by LOD/√2 in the remaining biomarkers, as previously described.^[^ ^45^ ^]^ In total, 75 biomarkers were obtained after QC.

### Ethical Approval Statement of Participants

The PROC study was approved by the Research Ethical Committee of Zhujiang Hospital, Southern Medical University (ethical approval no. 2019‐KY‐016‐02), and all participants gave their written informed consent to participate in the study. Human synovial fluid and tissues were obtained from patients with knee OA who underwent total knee arthroplasty in Nanjing Osteoarthritis Cohort (NOC), which was approved by the Ethical Committee of the Nanjing Drum Tower Hospital, the Affiliated Hospital of Nanjing University Medical School (ethical approval no. 2022‐176‐02). All procedures involving human participants were conducted in accordance with the Declaration of Helsinki and applicable regulations.

### Patient Consent Statement

Written informed consent was obtained from all participants after being informed.

### Statistical Analyses

The study includes all available samples (*N* = 207) of participants with knee synovitis and control individuals in the PROC study. The sample sizes are similar to those in previous publications.^[^ ^46^, ^47^ ^]^ During data collection for radiographic, metagenomic, metabolomics and proteomics analyses, investigators were blinded to group allocation.

The partial Spearman's rank correlation test^[^ ^48^ ^]^ was conducted to identify the correlations between multi‐omics features, as well as the correlation between Olink proteomics and the presence of synovitis. Further adjustment for the potential confounders, including age, gender, body mass index and Kellgren‐Lawrence grade, was conducted.^[^ ^49^, ^50^ ^]^ Four regular machine learning algorithms, including random forest, XGBoost, support vector machine (SVM) and k‐nearest neighbors (KNN) algorithms, were applied for model construction to further assess the predictive potential of multi‐omics features for synovitis diagnosis. These analyses were conducted on the screened microbial taxa, functional, metabolic and proteomic profiles, using the R package “mlr3.” To overcome performance bias and ensure a reliable selection of models, a nested cross‐validation approach was employed by both outer and inner loop serves.^[^ ^51^ ^]^ The outer loop serves to assess the quality of the model through 10‐fold cross‐validation. The optimal model was selected by the inner loop via leave‐one‐out cross‐validation (LOOCV). Discrimination of each model was quantitatively evaluated by the area under the curve (AUC) of the receiver operating characteristic (ROC).

All analyses were performed using R version 4.3.0 (R Foundation for Statistical Computing). Baseline tables were created with the use of the “compareGroups” package version 4.4.1. *P* < 0.05 was considered statistically significant. All Q values presented as FDR are *P* values corrected using the *p*.adjust function, and Q value < 0.2 was considered statistically significant.

The methodology was designed to adhere to the STROBE (Strengthening the Reporting of Observational Studies in Epidemiology) guidelines (Supplementary Materials and Methods), which were implemented to ensure the transparency and rigor of the observational research.

## Conflict of Interest

DJH serves as the editor for the osteoarthritis section in UpToDate and co‐Editor in Chief for the journal Osteoarthritis and Cartilage. DJH provides consulting advice on scientific advisory boards for Haleon, TLCBio, Novartis, Tissuegene, Sanofi, Enlivex. The other authors declare no competing interests.

## Author's Contribution

XS.W., Y.L., ZY.S., J.L., ZH.L., and JY.H. contributed equally to this work. All authors were involved in drafting the article or revising it critically for important intellectual content, and all authors approved the final version to be published. Prof. Ding had full access to all of the data in the study and took responsibility for the integrity of the data and the accuracy of the data analysis. The study was conceived and designed by XS.W., Z.A.L., DQ.S., and CH.D. Data were acquired by XS.W., ZY.S., J.L., JZ.R., JH.L., J.X., HT.S., TY.C., SF.L., ZH.Z., and ZB.W. Analysis and interpretation of the data were performed by XS.W., Y.L., ZY.S., ZH.L., JY.H., SX.H., PH.C., XL.C., SR.L., RS.T., and DJ.H.

## Supporting information



Supporting Information

Supporting Table

## Data Availability

The data that support the findings of this study are available from the corresponding author upon reasonable request.
