# Supplementary material for: Multi‐Omics Reveal the Dysregulated Gut‐Joint Axis in Knee Synovitis: Data from Two Osteoarthritis Studies in China
Source: Adv Sci (Weinh). 2025 Dec 7;13(7):e12020. doi: 10.1002/advs.202512020 (PMC12866873; doi:10.1002/advs.202512020)
Supplement: Supplementary file 1 — Supporting Information [file ADVS-13-e12020-s002.docx]

**Supplementary information**

**Multi-Omics Reveal the Dysregulated Gut-Joint Axis in Knee Synovitis: Data from Two Osteoarthritis Studies in China**

Xiaoshuai Wang *et al.*

Corresponding to Changhai Ding ([changhai.ding@utas.edu.au](mailto:changhai.ding@utas.edu.au))

**Table of contents**

[Supplementary Figures 3](#_Toc213446325)

[Supplementary Figure 1. Workflow and MRI detection for knee synovitis. 3](#_Toc213446326)

[Supplementary Figure 2. The abundance of *Bacteroidetes* depleted in knee synovitis at different levels. 4](#_Toc213446327)

[Supplementary Figure 3. Partial least squares-discriminant analysis (PLS-DA) of synovitis versus controls. 5](#_Toc213446328)

[Supplementary Figure 4. Serum metabolite of D-Galacturonic Acid and its links to gut microbial function. 6](#_Toc213446329)

[Supplementary Figure 5. Synovitis-related alterations in host plasma inflammatory proteins and their potential links to microbial function. 7](#_Toc213446330)

[Supplementary Figure 6. 2D intensity histogram of TWEAKR and PDPN/PCLAF/THY1 of mIHC in human synovium from patients with low-grade and high-grade synovitis, respectively. 8](#_Toc213446331)

[Supplementary Figure 7. Functional analysis for proteomics of synovial fluid in the Nanjing Osteoarthritis Cohort and prediction of potential drugs for TWEAKR. 9](#_Toc213446332)

[Supplementary Figure 8. The Receiver operating characteristic curves of random forest, XGBoost, support vector machine (SVM) and k-nearest neighbors (KNN) models were performed to evaluate the predictive performances for the presence of synovitis by individual omics datasets. 10](#_Toc213446333)

[Supplementary Materials and Methods 11](#_Toc213446334)

[*Radiographic acquisition and assessment* 11](#_Toc213446335)

[*Metagenomic analysis* 11](#_Toc213446336)

[*Ultra performance liquid chromatography/tandem mass spectrometry (UPLC-MS/MS) based Metabolomics of serum samples* 12](#_Toc213446337)

[*4D-DIA quantitative proteomics based on synovial fluid* 13](#_Toc213446338)

[*Single-cell RNA sequencing (scRNA-seq) data acquisition and analysis* 13](#_Toc213446339)

[*Histological analysis* 14](#_Toc213446340)

[*Multiplex immunohistochemistry (mIHC)* 14](#_Toc213446341)

[*Acquisition and Cell Culture of Human Fibroblast-like Synoviocytes (FLS)* 15](#_Toc213446342)

[*Western blotting* 15](#_Toc213446343)

[*Visualizations* 16](#_Toc213446344)

[Data availability 16](#_Toc213446345)

[Participants and public involvement 16](#_Toc213446346)

[Reference 16](#_Toc213446347)

# Supplementary Figures

**
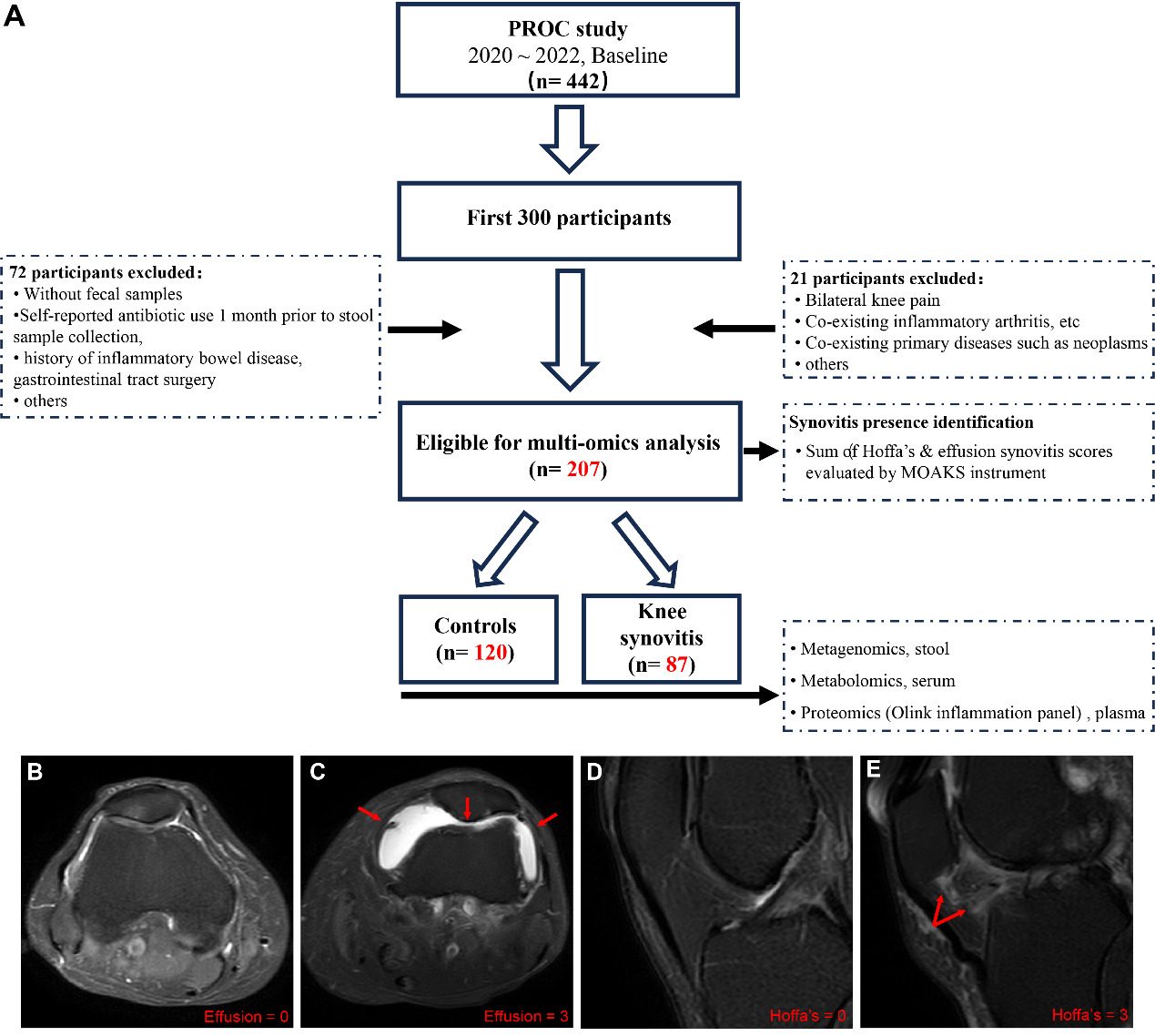
Supplementary Figure 1. Workflow and MRI detection for knee synovitis.**

(A) Workflow for sample groups and multi-omics data collection in synovitis from the PROC study; (B-E) Knee synovitis on Magnetic Resonance Imaging (MRI). Effusion-synovitis (axial, B-C) and Hoffa’s-synovitis scores (sagittal, D-E) were assessed by MRI Osteoarthritis Knee Score (MOAKS) system. For both, any finding (≥ 1), compared with no findings (= 0), was defined as presence of synovitis. Red arrows indicate severe lesions.

**
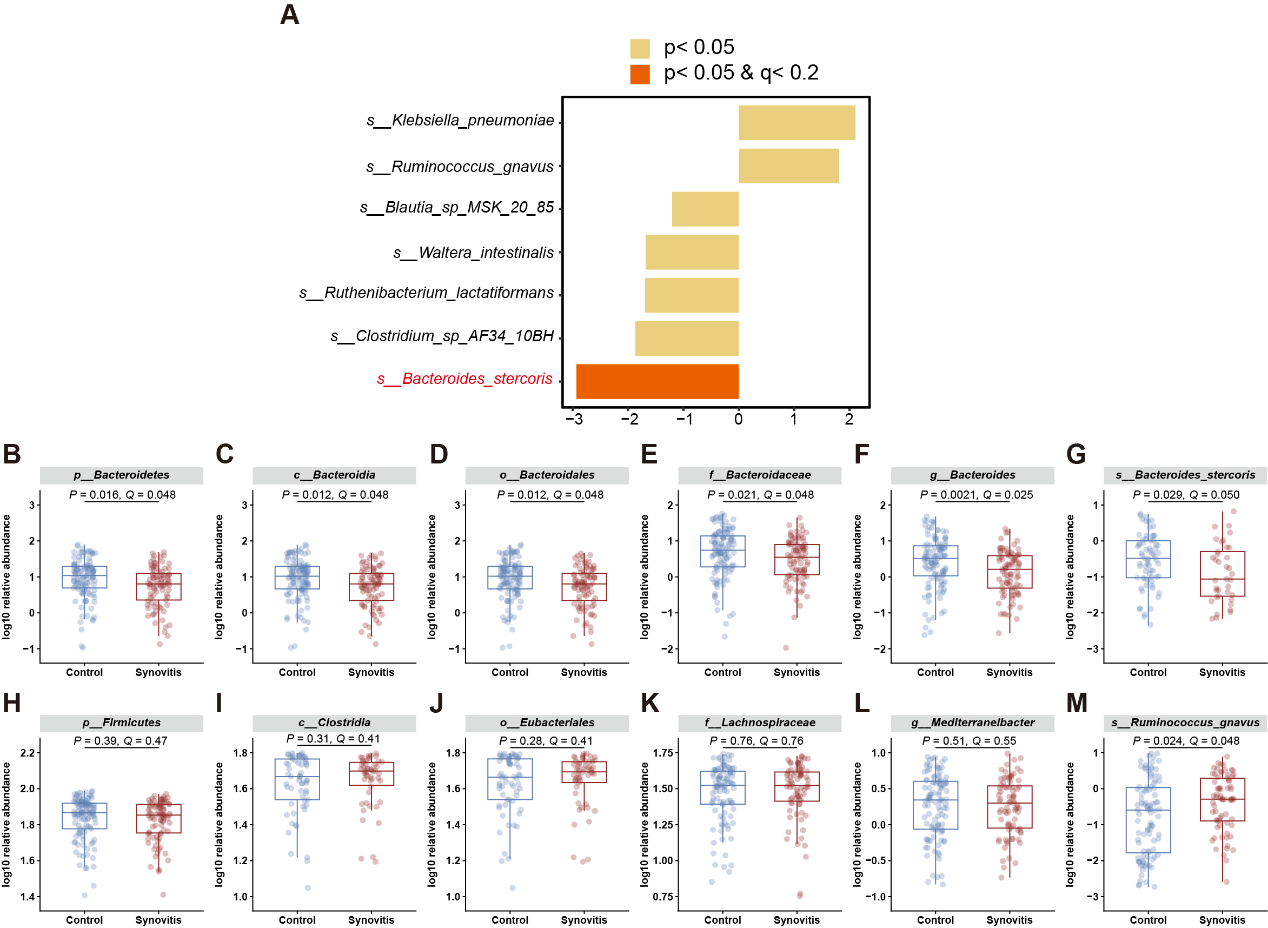
Supplementary Figure 2.** The abundance of *Bacteroidetes* depleted in knee synovitis at different levels**.**

(A) Bar plot illustrating association of gut microbial diversity at species level with synovitis by MaAsLin2 analysis, adjusted for potential confounders of age, gender, body mass index and Kellgren-Lawrence grade. Box plots comparing the difference in abundance of both *Bacteroides stercoris* (B-G) and *Ruminococcus* *gnavus* (H-M) at the phylum, class, order, family, genus and species levels, respectively (Wilcoxon rank-sum test with Benjamini-Hochberg correction, Q value, adjusted P value; Q < 0.2 is considered significant).

**
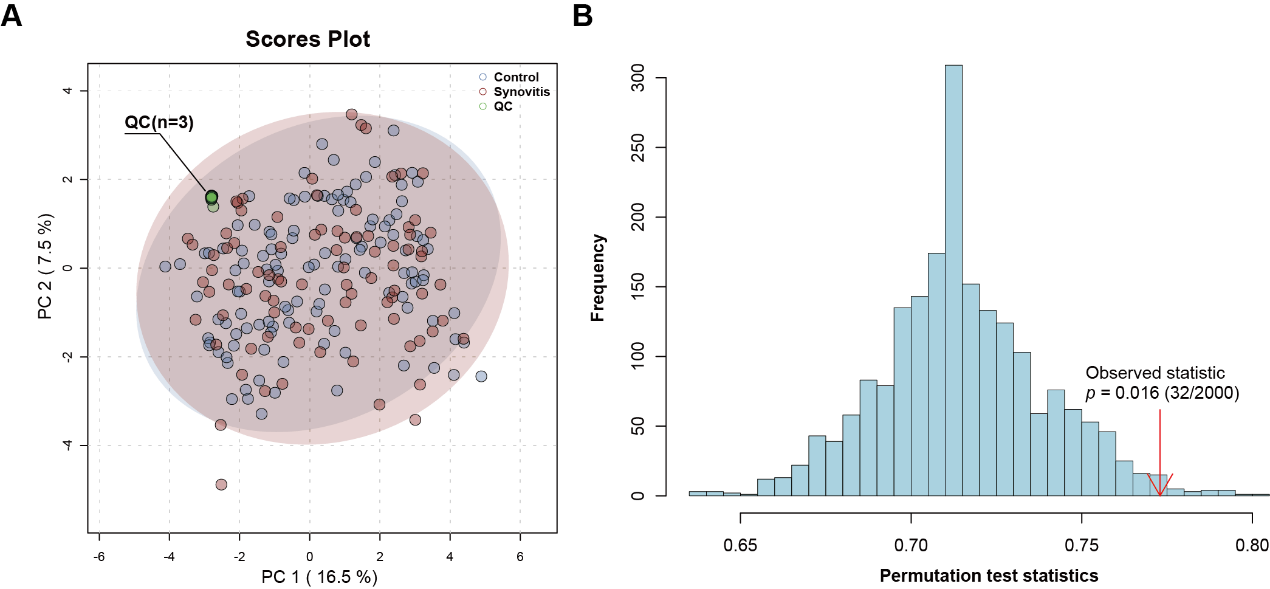
Supplementary Figure 3.** Partial least squares-discriminant analysis (PLS-DA) of synovitis versus controls**.**

(A) Scores plot illustrates 2 clusters from 3 kinds of sources (synovitis in red, control in blue, and quality control samples in green). The green dots depict a close clustering and being within two clusters, indicating the reliability of all samples. (B) The PLS-DA model was validated by permutation tests for 2,000 times based on separation distance of two different groups.

**
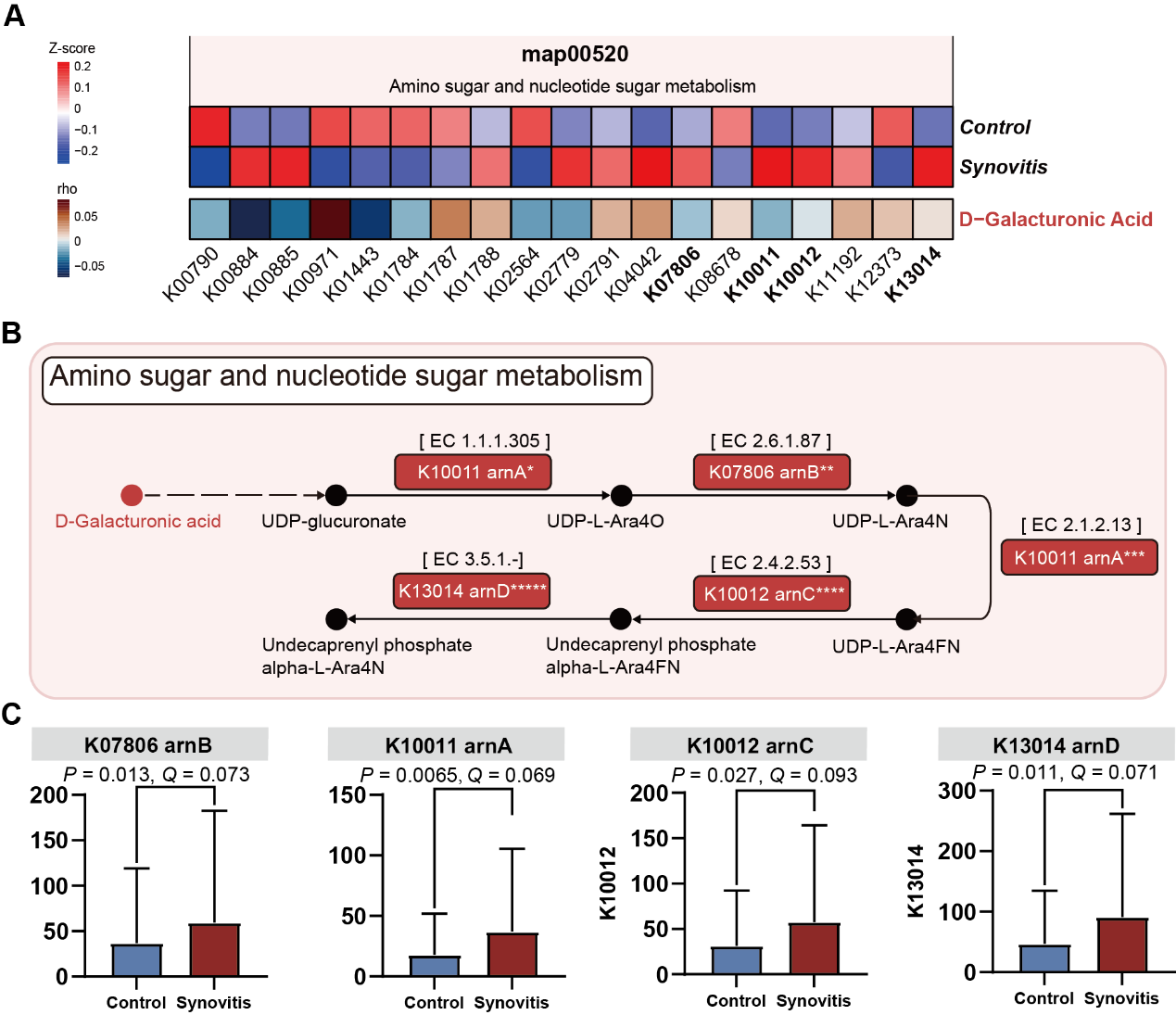
**Supplementary Figure 4. Serum metabolite of D-Galacturonic Acid and its links to gut microbial function.

(A) Heatmap displays the differential expression of KEGG orthologs (KOs) in synovitis and control groups (upper), and correlation between KOs and D-Galacturonic Acid in serum, within the pathway module modified from KEGG pathway maps “Amino sugar and nucleotide sugar metabolism” (partial Spearman, adjusted for age, gender, body mass index and Kellgren-Lawrence grade, Q value, adjusted P value; *Q < 0.2, **Q < 0.1, *** Q < 0.05, ****Q < 0.01;). (B) KO genes, represented as boxes and highlighted in red for elevation at synovitis group, are shown in the map00520 metabolic pathway modules, while red indicates upregulated metabolites in synovitis, and blue indicates the opposite. The pathway is generated on the basis of KEGG pathway maps. (C) The box plots show the log_10_ (relative abundances) of differential KOs between two groups (Wilcoxon rank sum test with Benjamini-Hochberg correction, fulfilling both P < 0.05 and Q < 0.2 is considered statistically significant). Asterisks represent different reactions according to KEGG: K10011 *arnA** for R07658; K07806 *arnB*** for R07659; K10011 *arnA**** for R07660; K10012 *arnC***** for R07661; K13014 *arnD****** for R07662.

**Supplementary
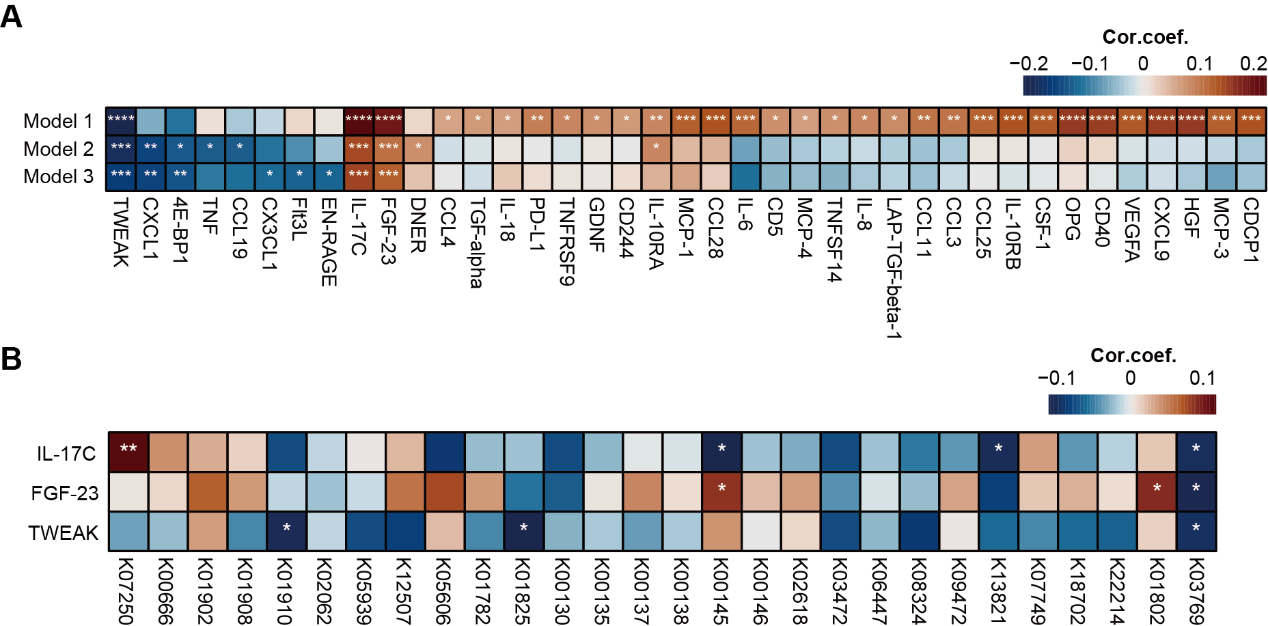
Figure 5.** Synovitis-related alterations in host plasma inflammatory proteins and their potential links to microbial function**.**

(A) Heatmap shows the association between all the significantly altered inflammatory proteins in plasma (at least in one model) and presence of synovitis. Asterisks indicate the statistical significance based on partial Spearman correlation. Model 1: crude model; model 2: adjusted for age, gender, body mass index; model 3: additional adjusted for Kellgren-Lawrence grade in model 2 (Q value, adjusted P value; *Q < 0.2, **Q < 0.1, ***Q < 0.05, ****Q < 0.01). (B) Heatmap shows the association between the significantly elevated inflammation-related proteins with the differing KOs of map00280 or map00907. Asterisks indicate the statistical significance based on partial Spearman correlation (adjusted for age, gender, body mass index and Kellgren-Lawrence grade, *Q < 0.2, **Q < 0.1, ***Q < 0.05, ****Q < 0.01).

**
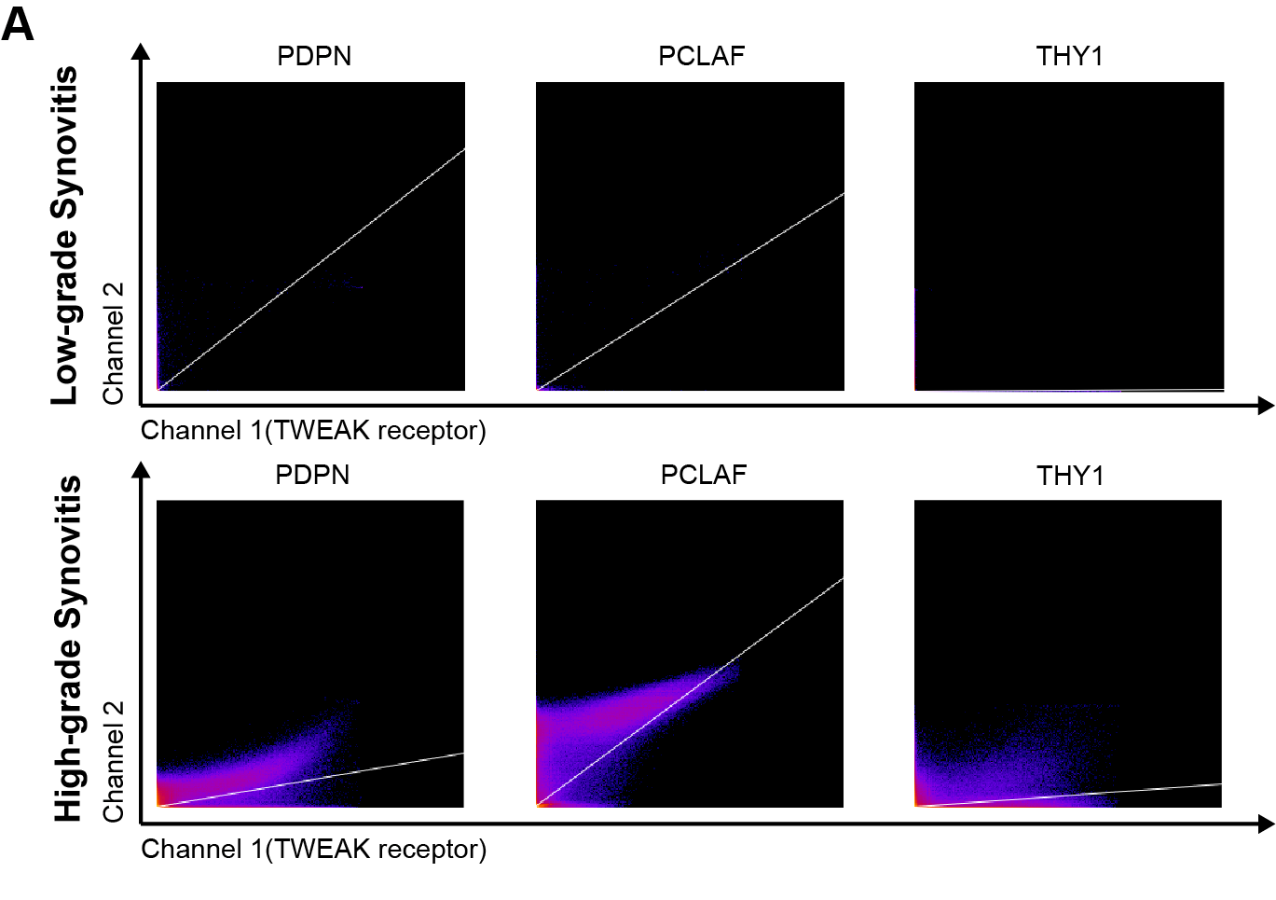
**Supplementary Figure 6. 2D intensity histogram of TWEAKR and PDPN/PCLAF/THY1 of mIHC in human synovium from patients with low-grade and high-grade synovitis, respectively.

**
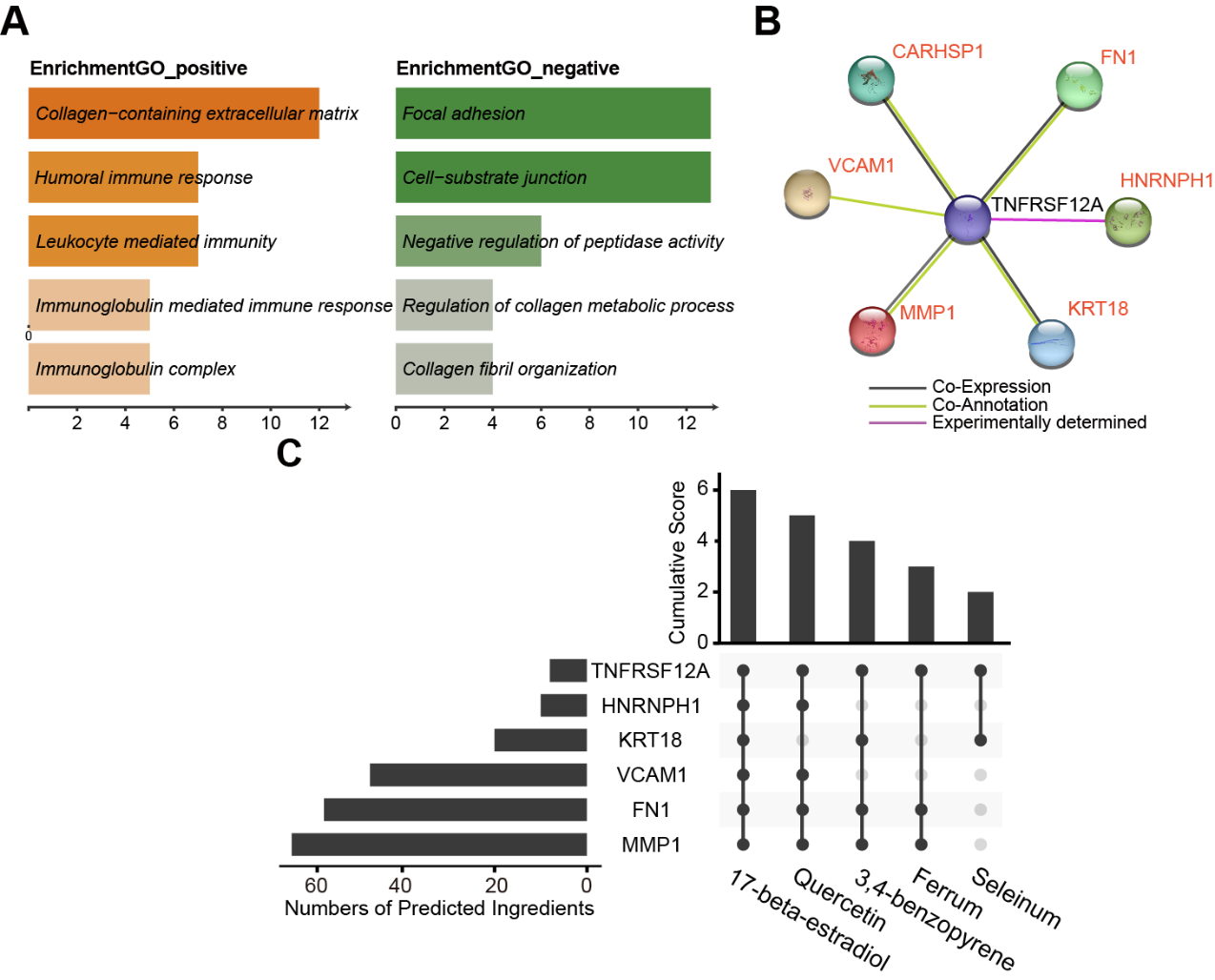
**Supplementary Figure 7. Functional analysis for proteomics of synovial fluid in the Nanjing Osteoarthritis Cohort and prediction of potential drugs for TWEAKR.

(A) Bar chart depicting Gene Ontology（GO）terms for proteins positively (left) or negatively correlated with high-grade synovitis, compared to low-grade synovitis. (B) Protein-protein interaction (PPI) network analysis was performed using STRING; predicted protein conformations are shown in the circular node of each protein. (C) UpSet plot exhibiting the cumulative scores of ingredients with potential therapeutic effects predicted by TWEAKR (TNFRSF12A) and its co-expressed proteins in Traditional Chinese Medical Syndrome Standardization Database (TCMSSD).

**
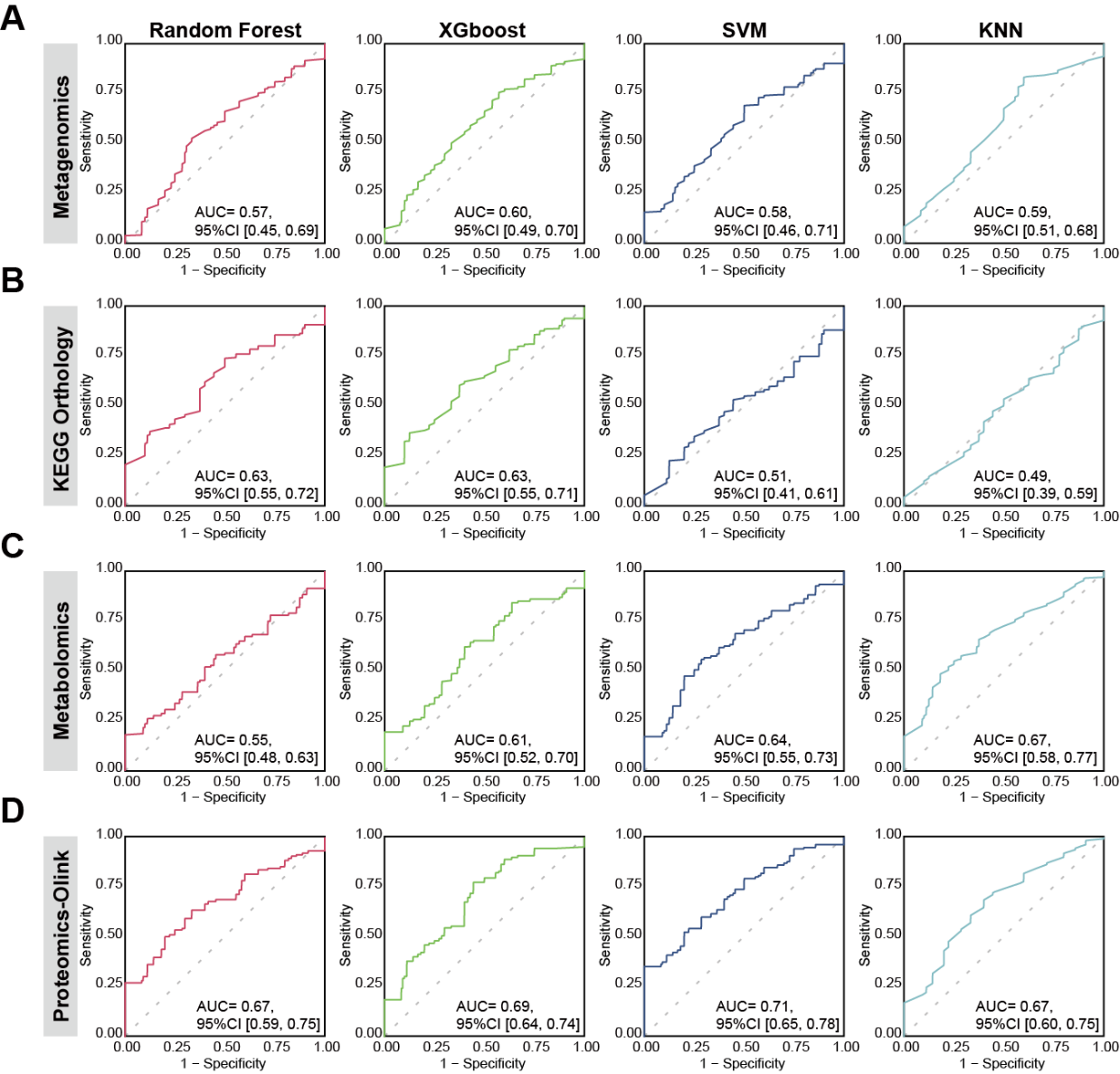
**Supplementary Figure 8. The Receiver operating characteristic curves of random forest, XGBoost, support vector machine (SVM) and k-nearest neighbors (KNN) models were performed to evaluate the predictive performances for the presence of synovitis by individual omics datasets.

(A) Gut microbiome profiles (Species level), (B) Gut microbial function (KOs), (C) Serum metabolites, and (D) Plasma inflammatory proteins.

**Supplementary Materials and Methods**

## *Radiographic acquisition and assessment*

Radiographs for knee joint were taken with the knee in an extended position and the patella in a central position. All radiographs were graded according to the Kellgren-Lawrence (KL) grade system ^[1]^.

## *Metagenomic analysis*

A total of 1743 taxa, including 1735 Bacteria, 6 Archaea and 2 Eukaryotes were identified. Since Bacteria were in the majority, including 13 phyla, 202 classes, 224 orders, 277 families, 865 genera, and 1735 species. The Alpha diversity (Shannon and Simpson index) was calculated for taxonomic profiles using the diversity function of “Vegan” package in R. The overall difference and similarity in bacterial communities between the synovitis and control groups (beta-diversity) were tested using the Bray-Curtis-based Permutational multivariate analysis of variance (PERMANOVA) by adonis2 function in “Vegan” package ^[2]^. To identify specific taxa significantly associated with knee synovitis, MaAsLin2 (Microbiome Multivariable Association with Linear Model 2) v1.10.0 was applied to count trends in abundance data. The execution parameters were set to: Analysis method = “LM”, normalization = “Default”, transform = “Default”, standardize = "TRUE", and correction = “BH”. To investigate the confounding effect, the fixed effects included the presence of synovitis, sex, age, body mass index and Kellgren-Lawrence grade ^[3]^. The functional annotation was conducted using the annotated pathways from the KEGG catalog (http://www.genome.ad.jp/kegg). The statistical significance of the observed differences between the synovitis and control groups was evaluated using *P* values that had been adjusted for multiple testing using the Benjamin and Hochberg false discovery rate method. The adjusted *P* values (Q values) were considered statistically significant if they were less than 0.2.

## *Ultra performance liquid chromatography/tandem mass spectrometry (UPLC-MS/MS) based Metabolomics of serum samples*

**Liquid samples class I:** serum samples were prepared, analyzed and processed using standard protocols. The samples which stored at -80 °C refrigerator were thawed on ice and vortexed for 10s. 50 μL of each sample and 300 μL of extraction solution (ACN: Methanol = 1:4, V/V) containing internal standards, were added into a 2 mL micro-centrifugetube. It was vortexed for 3 min and then centrifuged at 12000 revolution per minute (rpm) for 10 min (4 °C). 200 μL of the supernatant was collected and placed in -20 °C for 30 min, and then centrifuged at 12000 rpm for 3 min (4 °C as well). A 180 μL aliquots of supernatant were transferred as sample extract for LC-MS analysis^[4]^. **T3 UPLC Conditions:** The extracts of all sample were analyzed using an LC-ESI-MS/MS system (UPLC, ExionLC AD, <https://sciex.com.cn/>; MS, QTRAP® System, <https://sciex.com/>). The analytical conditions were as follows: a) UPLC: column, Waters ACQUITY UPLC HSS T3 C18 (1.8 µm, 2.1 mm*100 mm); b) column temperature, 40 °C; c) flow rate, 0.4 mL/min; d) injection volume, 2 μL; solvent system, e) water (0.1% formic acid): acetonitrile (0.1% formic acid); f) gradient program, 95:5 V/V at 0 min, 10:90 V/V at 11.0 min, 10:90 V/V at 12.0 min, 95:5 V/V at 12.1 min, 95:5 V/V at 14.0 min^[5]^. **ESI-QTRAP-MS/MS:** LIT and triple quadrupole (QQQ) scans were acquired on a triple quadrupole-linear ion trap mass spectrometer (QTRAP), QTRAP® LC-MS/MS System^[6]^, equipped with an ESI Turbo Ion-Spray interface, operating in positive and negative ion mode and controlled by Analyst 1.6.3 software (Sciex). The ESI source operation parameters were as follows: a) source temperature 500 °C; b) ion spray voltage (IS) 5500 V (positive), -4500 V (negative); c) ion source gas I (GSI), gas II (GSII), curtain gas (CUR) was set at 55, 60, and 25.0 psi, respectively; d) the collision gas (CAD) was high. Instrument tuning and mass calibration were performed with 10 and 100 μmol/L polypropylene glycol solutions in QQQ and LIT modes, respectively. A specific set of MRM transitions were monitored for each period according to the metabolites eluted within this period. **Quality control:** Data processing was performed using MetaboAnalyst v5.0 (https://www.metaboanalyst.ca/). The known metabolites were confirmed by ID at The Human Metabolome Database (HMDB). Data was filtered based on officially recommended rules (Between 500 and 1000 variables, 25% were filtered), following by log transformation^[7]^.

## *4D-DIA quantitative proteomics based on synovial fluid*

The 4D-DIA quantitative proteomics for synovial fluid was conducted by in Genechem Biotechnology Co., Ltd. (Shanghai, China). The experimental processes were performed according to the standardized method as previously described^[8]^: 1) Sample Preparation; 2) SDS-PAGE Separation; 3) Filter-aided sample preparation; 4) Peptide Fractionation with reversed phase (RP) Chromatography, and Spectral library generation. After that, raw data of DIA were obtained for subsequent analysis. Normalization was performance using the “Local Regression Normalization” since sample size is smaller than 500^[9]^.

The partial Spearman's rank correlation test (adjusted for age, gender, body mass index and Kellgren-Lawrence grade) was conducted to identify the correlation between a sum of 1140 proteins and high-grade (versus low-grade) synovitis. Proteins with estimated value of 0 or 1 were excluded, and a total of 1074 proteins were remained. The enriched list was obtained from protein with positive estimated value and the depleted list was the opposite, both with q-value cut-off to 0.2. Then, the upregulated and downregulated gene ontology (GO) terms analyses were performed accordingly, with the package clusterProfiler version 4.12.6, using the function enrichGO and setting the *p*-value cut-off to 0.05 and the q-value cut-off to 0.2. The Protein-protein interaction (PPI) networks of TNFRSF12A (TWEAK receptor) were assessed using STRING (Search Tool for the Retrieval of Interacting Genes/Proteins) (https://string-db.org), a database and visualization approach that integrates publicly available information sources^[10]^.

## *Single-cell RNA sequencing (scRNA-seq) data acquisition and analysis*

The ScRNA-seq (Chromium 10X) data for knee synovial tissue of knee OA was obtain publicly (GSE176308). The gene expression matrix files for low-grade synovitis (GSM5362559, early-stage OA) and high-grade synovitis (GSM5362561, late-stage OA) were chosen and reanalyzed ^[11]^. The cells exhibited gene numbers more than 200 and less than 6,000. Low quality cells (>25% mitochondrial genes) were abandoned and remaining cell counts were selected for subsequent analysis. Unsupervised clustering was conducted by principal component analysis (PCA) using identified highly variable genes, and visualization was performed in two dimensions by the Uniform Manifold Approximation and Projection (UMAP) method (Seurat ver. 4.4.0 software). The top 10 principal components were used in cell clustering with the resolutions set at 0.4

## *Histological analysis*

Human synovium specimens were fixed in 4% paraformaldehyde (PFA) for 48 h. They were then embedded in paraffin and sectioned into 4-μm-thick slices. Haematoxylin and eosin (H&E) staining were performed according to protocols of H&E Staining Kit (Beyotime). Synovitis scores were evaluated by reported scoring system ^[12]^.

## *Multiplex immunohistochemistry (mIHC)*

Synovium sections were de-paraffinized. And the sections were blocked with 3% H2O2 and 2% BSA after antigen retrieval. Different primary antibodies, anti-PCLAF (81533, Cell Signaling Technology), anti-TWEAK Receptor (27072, Cell Signaling Technology), anti-TYMS (15047, Proteintech), anti-PDGFRα (3174, Cell Signaling Technology) were sequentially used, followed by horseradish peroxidase conjugated secondary antibody incubation (F2761, F2765, T2769, and PA1-28565; Thermo Fisher Scientific) and tyramide signal amplification (TSA) [FITC-TSA, CY3-TSA, 594-TSA, and CY5-TSA (YB007, YOBIBIO)]. The nuclei were stained with 4,6-diamidino-2-phenylindole (DAPI; UBI5010, YOBIBIO). Multispectral images were obtained using a Pannoramic MIDI Scanner (3D HISTECH, Hungary). Multispectral images were analyzed, and positive cells were quantified at the single-cell level using the Caseviewer (version 2.3) image analysis software. The colocalization of TWEAKR with PDPN, PCLAF and THY1 was calculated using ImageJ software with “Coloc 2” plugin.  Three areas of each section were randomly selected to count the number of positive cells of TWEAKR and total cells, then calculate the positive cell rate and compare the rate between groups of high-grade and low-grade by un-paired t-test.

## *Acquisition and Cell Culture of Human Fibroblast-like Synoviocytes (FLS)*

Synovium was collected from OA patients receiving total knee replacement surgeries to isolate primary human FLSs. The obtained synovial tissue was subjected to enzymic digestion using 1 mg/ml Collagenase I (17100017, Gibco, USA) and 0.1 mg/ml DNase I (9003-98-9, Sigma-Aldrich, German) for 2 hours at 37 °C. The digestion buffer was then passed through a 100 µm filter (352360, Falcon, USA), and cells were collected using centrifugation at 500 × g for 5 minutes. The cells were cultured in DMEM/F12 Medium (A4192001, Gibco, USA) supplemented with 10% FBS (16000044, Gibco, USA) and 1% penicillin‐streptomycin (15140122, Gibco, USA). For experiments, human FLSs were cultured for a minimum of 6 hours in serum-free DMEM/F12 Medium, followed by stimulation with concentration of 0/100/200 ng/ml TWEAK recombinant protein (HY-PT7309, MCE, China) for 24 or 48 hours, respectively.

## *Western blotting*

Total protein was extracted using RIPA Lysis Buffer (R0010, Solarbio) with 1 mmol/L phenylmethanesulfonyl fluoride (329-98-6, Solarbio) and 1 mmol/L phosphatase inhibitor cocktail (B15002, Bimake, USA). Nuclear and cytoplasmic proteins were extracted using a Nucleoplasmic Protein Extraction Kit (R0050, Solarbio), and protein concentrations were measured by the BCA Assay Kit (23225, Thermo Scientific, USA). A 10% (w/v) SDS-polyacrylamide gel was used to separate proteins (PG112, EpiZyme, China), which were transferred onto polyvinylidene fluoride membranes (IPVH00010, Millipore, USA). After blocking with 5% (w/v) milk (1172GR500, Biofroxx) for 1 h at 37 °C, the membrane was incubated with primary antibodies of TWEAKR (27072, CST, USA) and beta-actin (4967, CST, USA) overnight at 4 °C. A horseradish peroxidase-conjugated goat anti-rabbit/mouse IgG (BL003A or BL001A, Biosharp) was used as a secondary antibody. All images were obtained using a Western Blotting Imaging System (Tanon, China).

## *Visualizations*

The ggplot2 v3.5.1, ggpubr v0.6.0, patchwork v1.2.0, ggthemes v5.1.0, pheatmap v1.0.12, ggalluvial v0.12.5 in R were used.

# Data availability

The raw metagenomic sequence data are available upon request from the corresponding author Prof. Changhai Ding (Changhai.Ding@utas.edu.au). The publicly available single-cell RNA-seq data with the accession ID GSE176308 are at the NCBI’s Gene Expression Omnibus (GEO) data repository.

# Participants and public involvement

This research was done without participant and public involvement. Participants and public were not invited to comment on the study design and were not consulted to develop participant relevant outcomes or interpret the results.

# Reference

[1] Schiphof D, Boers M, Bierma-Zeinstra SM. Differences in descriptions of Kellgren and Lawrence grades of knee osteoarthritis. Ann Rheum Dis. 2008;67(7):1034-1036. doi:10.1136/ard.2007.079020
[2] Yang J, Zheng P, Li Y, et al. Landscapes of bacterial and metabolic signatures and their interaction in major depressive disorders. Sci Adv. 2020;6(49):eaba8555. Published 2020 Dec 2. doi:10.1126/sciadv.aba8555
[3] Ferreiro AL, Choi J, Ryou J, et al. Gut microbiome composition may be an indicator of preclinical Alzheimer's disease. Sci Transl Med. 2023;15(700):eabo2984. doi:10.1126/scitranslmed.abo2984
[4] Li M, Haixia Y, Kang M, et al. The Arachidonic Acid Metabolism Mechanism Based on UPLC-MS/MS Metabolomics in Recurrent Spontaneous Abortion Rats. Front Endocrinol (Lausanne). 2021;12:652807. Published 2021 Apr 2. doi:10.3389/fendo.2021.652807
[5] Chen Y, Zhang R, Song Y, et al. RRLC-MS/MS-based metabonomics combined with in-depth analysis of metabolic correlation network: finding potential biomarkers for breast cancer. Analyst. 2009;134(10):2003-2011. doi:10.1039/b907243h
[6] Crescenzi MA, D'Urso G, Piacente S, Montoro P. A Comparative UHPLC-Q-Trap-MS/MS-Based Metabolomics Analysis to Distinguish Foeniculum vulgare Cultivars' Antioxidant Extracts. Molecules. 2023;28(2):900. Published 2023 Jan 16. doi:10.3390/molecules28020900
[7] Pang Z, Zhou G, Ewald J, et al. Using MetaboAnalyst 5.0 for LC-HRMS spectra processing, multi-omics integration and covariate adjustment of global metabolomics data. Nat Protoc. 2022;17(8):1735-1761. doi:10.1038/s41596-022-00710-w
[8] Huang Z, Deng C, Ma C, et al. Identification and validation of the surface proteins FIBG, PDGF-β, and TGF-β on serum extracellular vesicles for non-invasive detection of colorectal cancer: experimental study. Int J Surg. 2024;110(8):4672-4687. Published 2024 Aug 1. doi:10.1097/JS9.0000000000001533
[9] Callister SJ, Barry RC, Adkins JN, et al. Normalization approaches for removing systematic biases associated with mass spectrometry and label-free proteomics. J Proteome Res. 2006;5(2):277-286. doi:10.1021/pr050300l
[10] Duggan MR, Butler L, Peng Z, et al. Plasma proteins related to inflammatory diet predict future cognitive impairment [published correction appears in Mol Psychiatry. 2023 Apr;28(4):1610. doi: 10.1038/s41380-023-02007-0.]. Mol Psychiatry. 2023;28(4):1599-1609. doi:10.1038/s41380-023-01975-7
[11] Nanus DE, Badoume A, Wijesinghe SN, et al. Synovial tissue from sites of joint pain in knee osteoarthritis patients exhibits a differential phenotype with distinct fibroblast subsets. EBioMedicine. 2021;72:103618. doi:10.1016/j.ebiom.2021.103618
[12] Krenn V, Morawietz L, Burmester GR, et al. Synovitis score: discrimination between chronic low-grade and high-grade synovitis. Histopathology. 2006;49(4):358-364. doi:10.1111/j.1365-2559.2006.02508.x
